# Diagnosis of Multisystem Inflammatory Syndrome in Children by a Whole-Blood Transcriptional Signature

**DOI:** 10.1093/jpids/piad035

**Published:** 2023-05-31

**Authors:** Heather R Jackson, Luca Miglietta, Dominic Habgood-Coote, Giselle D’Souza, Priyen Shah, Samuel Nichols, Ortensia Vito, Oliver Powell, Maisey Salina Davidson, Chisato Shimizu, Philipp K A Agyeman, Coco R Beudeker, Karen Brengel-Pesce, Enitan D Carrol, Michael J Carter, Tisham De, Irini Eleftheriou, Marieke Emonts, Cristina Epalza, Pantelis Georgiou, Ronald De Groot, Katy Fidler, Colin Fink, Daniëlle van Keulen, Taco Kuijpers, Henriette Moll, Irene Papatheodorou, Stephane Paulus, Marko Pokorn, Andrew J Pollard, Irene Rivero-Calle, Pablo Rojo, Fatou Secka, Luregn J Schlapbach, Adriana H Tremoulet, Maria Tsolia, Effua Usuf, Michiel Van Der Flier, Ulrich Von Both, Clementien Vermont, Shunmay Yeung, Dace Zavadska, Werner Zenz, Lachlan J M Coin, Aubrey Cunnington, Jane C Burns, Victoria Wright, Federico Martinon-Torres, Jethro A Herberg, Jesus Rodriguez-Manzano, Myrsini Kaforou, Michael Levin

**Affiliations:** Department of Infectious Disease, Faculty of Medicine, Imperial College London, London, UK; Centre for Paediatrics and Child Health, Imperial College London, London, SW7 2AZ, UK; Department of Infectious Disease, Faculty of Medicine, Imperial College London, London, UK; Department of Electrical and Electronic Engineering, Faculty of Engineering, Imperial College London, London, UK; Department of Infectious Disease, Faculty of Medicine, Imperial College London, London, UK; Centre for Paediatrics and Child Health, Imperial College London, London, SW7 2AZ, UK; Department of Infectious Disease, Faculty of Medicine, Imperial College London, London, UK; Centre for Paediatrics and Child Health, Imperial College London, London, SW7 2AZ, UK; Department of Infectious Disease, Faculty of Medicine, Imperial College London, London, UK; Centre for Paediatrics and Child Health, Imperial College London, London, SW7 2AZ, UK; Department of Infectious Disease, Faculty of Medicine, Imperial College London, London, UK; Centre for Paediatrics and Child Health, Imperial College London, London, SW7 2AZ, UK; Department of Infectious Disease, Faculty of Medicine, Imperial College London, London, UK; Centre for Paediatrics and Child Health, Imperial College London, London, SW7 2AZ, UK; Department of Infectious Disease, Faculty of Medicine, Imperial College London, London, UK; Centre for Paediatrics and Child Health, Imperial College London, London, SW7 2AZ, UK; Department of Infectious Disease, Faculty of Medicine, Imperial College London, London, UK; Centre for Paediatrics and Child Health, Imperial College London, London, SW7 2AZ, UK; Department of Pediatrics, Rady Children’s Hospital and University of California San Diego, La Jolla, California, USA; Department of Pediatrics, Inselspital, Bern University Hospital, University of Bern, Bern, Switzerland; Department of Paediatric Infectious Diseases and Immunology, Wilhelmina Children’s Hospital, University Medical Centre Utrecht, Utrecht, The Netherlands; Joint Research Unit Hospices Civils de Lyon-bioMérieux, Lyon Sud Hospital, Pierre-Bénite, France; Department of Clinical Infection Microbiology and Immunology, University of Liverpool Institute of Infection, Veterinary and Ecological Sciences, Liverpool, UK; Paediatric Intensive Care, Evelina London Children’s Hospital, Guy’s and St Thomas’ NHS Foundation Trust, London, UK; Department of Women and Children’s Health, School of Life Course Sciences, King’s College London, St Thomas’ Hospital, London, UK; Department of Infectious Disease, Faculty of Medicine, Imperial College London, London, UK; Centre for Paediatrics and Child Health, Imperial College London, London, SW7 2AZ, UK; Second Department of Paediatrics, National and Kapodistrian University of Athens (NKUA), School of Medicine, P. and A. Kyriakou Children’s Hospital, Athens, Greece; Translational and Clinical Research Institute, Newcastle University, Newcastle upon Tyne, UK; Paediatric Infectious Diseases and Immunology Department, Newcastle upon Tyne Hospitals Foundation Trust, Great North Children’s Hospital, Newcastle upon Tyne, UK; NIHR Newcastle Biomedical Research Centre, Newcastle upon Tyne Hospitals NHS Trust and Newcastle University, Newcastle upon Tyne, UK; Pediatric Infectious Diseases Unit, Pediatric Department, Hospital Doce de Octubre, Madrid, Spain; Department of Electrical and Electronic Engineering, Faculty of Engineering, Imperial College London, London, UK; Department of Pediatrics, Division of Pediatric Infectious Diseases and Immunology and Laboratory of Infectious Diseases, Radboud Institute of Molecular Life Sciences, Radboudumc, Nijmegen, The Netherlands; Academic Department of Paediatrics, Royal Alexandra Children’s Hospital, University Hospitals Sussex, Brighton, UK; Micropathology Ltd., University of Warwick, Warwick, UK; SkylineDx, Rotterdam, The Netherlands; Department of Pediatric Immunology, Rheumatology, and Infectious Diseases, Emma Children’s Hospital, Amsterdam University Medical Centre, Amsterdam, The Netherlands; Sanquin Research, Department of Blood Cell Research, and Landsteiner Laboratory, Amsterdam University Medical Centre, Amsterdam, The Netherlands; Department of Pediatrics, Erasmus MC Sophia Children’s Hospital, Rotterdam, The Netherlands; Gene Expression Team, European Molecular Biology Laboratory, EMBL-European Bioinformatics Institute (EMBL-EBI), Hinxton, Cambridge, UK; Oxford Vaccine Group, Department of Paediatrics, University of Oxford and the NIHR Oxford Biomedical Research Centre, Oxford, UK; Division of Pediatrics, University Medical Centre Ljubljana and Medical Faculty, University of Ljubljana, Ljubljana, Slovenia; Oxford Vaccine Group, Department of Paediatrics, University of Oxford and the NIHR Oxford Biomedical Research Centre, Oxford, UK; Pediatrics Department, Translational Pediatrics and Infectious Diseases Section, Santiago de Compostela, Spain; Genetics–Vaccines–Infectious Diseases and Pediatrics Research Group GENVIP, Instituto de Investigación Sanitaria de Santiago (IDIS), Universidade de Santiago de Compostela (USC), Santiago de Compostela, Spain; Unidade de Xenética, Departamento de Anatomía Patolóxica e Ciencias Forenses, Instituto de Ciencias Forenses, Facultade de Medicina, Universidade de Santiago de Compostela, Galicia, Spain; GenPoB Research Group, Instituto de Investigaciones Sanitarias (IDIS), Hospital Clínico Universitario de Santiago (SERGAS), Galicia, Spain; Pediatric Infectious Diseases Unit, Pediatric Department, Hospital Doce de Octubre, Madrid, Spain; Medical Research Council Unit, The Gambia at the London School of Hygiene and Tropical Medicine, Banjul, Gambia; Department of Intensive Care and Neonatology, and Children’s Research Center, University Children`s Hospital Zurich, Zurich, Switzerland; Child Health Research Centre, The University of Queensland, Brisbane, Queensland, Australia; Department of Pediatrics, Rady Children’s Hospital and University of California San Diego, La Jolla, California, USA; Second Department of Paediatrics, National and Kapodistrian University of Athens (NKUA), School of Medicine, P. and A. Kyriakou Children’s Hospital, Athens, Greece; Medical Research Council Unit, The Gambia at the London School of Hygiene and Tropical Medicine, Banjul, Gambia; Department of Paediatric Infectious Diseases and Immunology, Wilhelmina Children’s Hospital, University Medical Centre Utrecht, Utrecht, The Netherlands; Division of Pediatric Infectious Diseases, Department of Pediatrics, Dr von Hauner Children’s Hospital, University Hospital, LMU Munich, Munich, Germany; Department of Paediatric Infectious Diseases and Immunology, Erasmus MC Sophia Children’s Hospital, Rotterdam, The Netherlands; Clinical Research Department, Faculty of Infectious and Tropical Disease, London School of Hygiene and Tropical Medicine, London, UK; Department of Pediatrics, Children’s Clinical University Hospital, Rīga, Latvia; Department of General Paediatrics, University Clinic of Paediatrics and Adolescent Medicine, Medical University Graz, Austria; Department of Microbiology and Immunology, University of Melbourne at The Peter Doherty Institute for Infection and Immunity, Melbourne, Australia; Department of Infectious Disease, Faculty of Medicine, Imperial College London, London, UK; Centre for Paediatrics and Child Health, Imperial College London, London, SW7 2AZ, UK; Department of Pediatrics, Rady Children’s Hospital and University of California San Diego, La Jolla, California, USA; Department of Infectious Disease, Faculty of Medicine, Imperial College London, London, UK; Centre for Paediatrics and Child Health, Imperial College London, London, SW7 2AZ, UK; Pediatrics Department, Translational Pediatrics and Infectious Diseases Section, Santiago de Compostela, Spain; Genetics–Vaccines–Infectious Diseases and Pediatrics Research Group GENVIP, Instituto de Investigación Sanitaria de Santiago (IDIS), Universidade de Santiago de Compostela (USC), Santiago de Compostela, Spain; Unidade de Xenética, Departamento de Anatomía Patolóxica e Ciencias Forenses, Instituto de Ciencias Forenses, Facultade de Medicina, Universidade de Santiago de Compostela, Galicia, Spain; GenPoB Research Group, Instituto de Investigaciones Sanitarias (IDIS), Hospital Clínico Universitario de Santiago (SERGAS), Galicia, Spain; Department of Infectious Disease, Faculty of Medicine, Imperial College London, London, UK; Centre for Paediatrics and Child Health, Imperial College London, London, SW7 2AZ, UK; Department of Infectious Disease, Faculty of Medicine, Imperial College London, London, UK; Department of Infectious Disease, Faculty of Medicine, Imperial College London, London, UK; Centre for Paediatrics and Child Health, Imperial College London, London, SW7 2AZ, UK; Department of Infectious Disease, Faculty of Medicine, Imperial College London, London, UK; Centre for Paediatrics and Child Health, Imperial College London, London, SW7 2AZ, UK

**Keywords:** COVID-19, diagnostic signature, host diagnostics, host response, MIS-C, pediatric infectious diseases, rapid diagnostics, transcriptomics

## Abstract

**Background:**

To identify a diagnostic blood transcriptomic signature that distinguishes multisystem inflammatory syndrome in children (MIS-C) from Kawasaki disease (KD), bacterial infections, and viral infections.

**Methods:**

Children presenting with MIS-C to participating hospitals in the United Kingdom and the European Union between April 2020 and April 2021 were prospectively recruited. Whole-blood RNA Sequencing was performed, contrasting the transcriptomes of children with MIS-C (*n* = 38) to those from children with KD (*n =* 136), definite bacterial (DB; *n =* 188) and viral infections (DV; *n =* 138). Genes significantly differentially expressed (SDE) between MIS-C and comparator groups were identified. Feature selection was used to identify genes that optimally distinguish MIS-C from other diseases, which were subsequently translated into RT-qPCR assays and evaluated in an independent validation set comprising MIS-C (*n =* 37), KD (*n =* 19), DB (*n =* 56), DV (*n =* 43), and COVID-19 (*n =* 39).

**Results:**

In the discovery set, 5696 genes were SDE between MIS-C and combined comparator disease groups. Five genes were identified as potential MIS-C diagnostic biomarkers (*HSPBAP1*, *VPS37C*, *TGFB1*, *MX2*, and *TRBV11-2*), achieving an AUC of 96.8% (95% CI: 94.6%–98.9%) in the discovery set, and were translated into RT-qPCR assays. The RT-qPCR 5-gene signature achieved an AUC of 93.2% (95% CI: 88.3%–97.7%) in the independent validation set when distinguishing MIS-C from KD, DB, and DV.

**Conclusions:**

MIS-C can be distinguished from KD, DB, and DV groups using a 5-gene blood RNA expression signature. The small number of genes in the signature and good performance in both discovery and validation sets should enable the development of a diagnostic test for MIS-C.

## INTRODUCTION

Since its recognition in 2020, multisystem inflammatory syndrome in children (MIS-C) temporally associated with SARS-CoV-2 infection has emerged as an important cause of critical illness in children worldwide [1–3]. MIS-C occurs 2–6 weeks after SARS-CoV-2 infection [1–3] and is characterized by persistent fever and nonspecific symptoms, particularly abdominal pain, vomiting, headache, and fatigue [3–5]. Conjunctival injection and rash resembling Kawasaki disease (KD) occur in many patients [2, 6, 7]. Severely affected children often develop shock and multi-organ failure [3, 4]. Laboratory studies show increased inflammation with elevated C-reactive protein (CRP), ferritin, troponin, and brain natriuretic peptide, and reduced hemoglobin, platelets, and lymphocytes [3].

MIS-C diagnosis is based on clinical criteria established by consensus in the early months after the first recognition of the disorder. Diagnostic criteria from the WHO [8], Royal College of Paediatrics (United Kingdom) [9] and CDC (United States) [10] are largely overlapping and require the presence of fever, multisystem organ involvement, laboratory evidence of inflammation, and exclusion of other infectious and inflammatory disorders. The CDC and WHO definitions also include evidence of recent SARS-CoV-2 infection or exposure.

While the rapidly developed case definitions for MIS-C have provided clinicians with valuable tools for the recognition of this new disorder, these were created by expert opinion after the initial cluster of less than 60 patients [3]. Until now, a major difficulty facing clinicians in the diagnosis and management of MIS-C has been the lack of a reliable diagnostic test. The clinical features of MIS-C are nonspecific and overlap with those of many childhood infectious and inflammatory diseases, including sepsis, severe bacterial and viral infections, KD, staphylococcal and streptococcal toxic shock syndromes, gastrointestinal infections, appendicitis, systemic juvenile rheumatoid arthritis, macrophage activation syndrome, and hemophagocytic lymphohistiocytosis (HLH) [11–13]. Due to these diagnostic difficulties, patients are often treated with prolonged courses of antibiotics while awaiting cultures to exclude bacterial infection before the diagnosis of MIS-C is considered. Conversely, the similarity in features with disorders such as HLH and KD has led to several immunomodulatory agents typically used in these other conditions being administered to children with MIS-C with no clear evidence yet of the optimal treatment [14, 15]. Thus, there is an urgent need for a diagnostic test to distinguish MIS-C from other pediatric infectious and inflammatory disorders.

A growing body of research suggests that individual infectious and inflammatory diseases are characterized by unique patterns of host RNA abundance in whole blood. Sparse gene signatures, based on small numbers of transcripts, have been reported for several diseases including tuberculosis disease [16–18], malaria [19], bacterial and viral infections [20, 21], and KD [22]. MIS-C has already been shown to elicit specific changes in gene expression compared to healthy controls and pediatric COVID-19 using targeted and untargeted transcriptomic approaches, respectively [23, 24]. We show here that MIS-C can be distinguished from KD and a wide range of bacterial and viral infections by a sparse RNA signature that could form the basis of a diagnostic test for clinical use.

## METHODS

### Clinical Cohorts and Study Design

At the onset of the COVID-19 pandemic, the authors had ongoing patient recruitment to successive European Union-funded international studies: DIAMONDS (Diagnosis and Management of Febrile Illness using RNA Personalised Molecular Signature Diagnosis) and PERFORM (Personalised Risk Assessment in Febrile Illness to Optimise Real-life Management across the European Union). Samples and data were also available from studies recruiting before the start of the COVID-19 pandemic: EUCLIDS (European Union Childhood Life-Threatening Infectious Disease Study) and Kawasaki Disease Research Centre Study at the University of California San Diego (UCSD). Further details of each cohort are provided in Supplementary Methods.

Patients recruited for each study were phenotyped according to our published algorithm (Supplementary Methods) [20, 25, 26]. KD was diagnosed on the basis of the American Heart Association diagnostic criteria [27], and MIS-C was diagnosed based on the WHO criteria [8]. In both discovery and validation sets, we included patients with MIS-C, KD, confirmed bacterial infection (termed definite bacterial; DB), and confirmed viral infections (definite viral; DV). Healthy control children were included but only used to correct batch effects between groups. The DB group mainly included patients in whom an appropriate bacterial pathogen was isolated from a normally sterile site. However, we also included a group of pathogens which are normally identified only on mucosal surfaces or by nonculture methods, such as *Campylobacter*, *Salmonella*, *Borrelia burgdorferi*, and *Bordetella pertussis.* We termed these nonsterile site definite bacterial infections (NSDB), which we considered important to include as a comparator due to the predominance of gastrointestinal symptoms in MIS-C cases, resembling enteric infections and the breadth of multisystem symptoms. DV was conditional upon the identification of a virus compatible with the clinical syndrome with no evidence of bacterial infection and CRP ≤ 60 mg/L. In the validation set, we additionally included children with COVID-19. Due to the broad range of presenting symptoms of MIS-C [28], we purposefully included various bacterial and viral pathogens to compare to MIS-C with the ultimate aim of discovering a signature that was robust to clinical presentation and/or pathogen type. The signature discovery was performed using a discovery set generated through RNA Sequencing (RNA-Seq). Signature validation was carried out using an independent validation set generated by reverse transcription-quantitative polymerase chain reaction (RT-qPCR).

### Study Oversight and Ethics

Patients were recruited according to the approved enrollment procedures of each study and with the informed consent of parents and assent for older children. Each study was approved by the relevant institutional and national research ethics committees: DIAMONDS (London—Dulwich Research Ethics Committee: 20/HRA/1714); PERFORM (London—Central Research Ethics Committee: 16/LO/1684); and EUCLIDS (NRES Committee London—Fulham: 11/LO/1982) studies. The UCSD KD study was approved by the UCSD Investigational Review Board (UCSD [Human Research Protection Program 140220]).

### Signature Discovery Stage

#### Sample Collection and RNA Sequencing.

Venous blood was collected from patients at the earliest time point after recruitment and prior to treatment with IVIG or immunomodulator into PAXgene Blood RNA (PreAnalytiX, Germany) and stored at –80°C until extraction. Total RNA was isolated using a recommended methodology (including miRNA for PAXgene blood RNA vacutainers), and after additional DNAse treatment was sent for RNA-Seq at The Wellcome Centre for Human Genetics in Oxford, United Kingdom, using a Novaseq6000 platform at 150 bp paired-end configuration, generating a raw read count of 30 million reads per sample.

#### Analysis of RNA Sequencing Data and Discovery of Diagnostic Gene Signature.

All statistical analyses were performed using the statistical software R (R version 4.0.3) [29]. Full details of the normalization and quality control (QC) methods for the RNA-Seq data are in the Supplementary Methods. Healthy pediatric controls included in the RNA-Seq experiment were used for batch normalization. After QC, genes significantly differentially expressed (SDE) between MIS-C and each of the individual disease groups (KD, DV, and DB), as well as the groups combined were identified using DESeq2 [30], with age, sex and RNA-Seq dataset included in the DESeq2 model. Genes with Benjamini–Hochberg [31] adjusted *P* values <.05 were considered significantly differentially expressed (SDE). Genes with adjusted *P* values <.001, absolute log fold-change (LFC) values >0.5, and with mean gene count >50 in all disease groups and >100 in at least 1 disease group were taken forward to variable selection using a modified version of Forward Selection-Partial Least Squares (FS-PLS) [17, 20, 32]. The modified version of FS-PLS enables multiple comparisons to be considered in parallel [32] with details of the comparisons considered in the Supplementary Methods. The performance of the combined FS-PLS set and the top SDE gene between MIS-C and all other disease groups was evaluated using the area under the receiver operating characteristic (ROC) curve (AUC) calculated using a weighted disease risk score (DRS) for each individual calculated from the combined information of all transcripts in the model (Supplementary Methods). The weighted DRS is an adaptation of the DRS approach described in [18, 20, 21]. The optimal weighted DRS threshold for MIS-C classification in the discovery set was selected using Youden’s index [33] which optimizes the trade-off between sensitivity and specificity to maximize AUC.

### Signature Validation Stage

#### Translation to RT-qPCR Assays.

Genes identified in the discovery phase were considered for validation by RT-qPCR, in addition to a reference gene—Glyceraldehyde 3-phosphate dehydrogenase (*GAPDH*)—which was used to normalize the RNA input of each sample. Primers were designed as described in Supplementary Table 2. The validation was performed using the Biomark HD (Fluidigm) and the 192.24 Dynamic Array integrated fluidic circuit (IFC) following manufacturer instructions. The IFC required the following 3 steps: reverse transcription, pre-amplification, and RT-qPCR (Supplementary Methods).

#### Statistical Analysis of RT-qPCR Data.

Following QC and normalization of the RT-qPCR data (Supplementary Methods), the performance of the signature was evaluated. Weighted DRS were calculated for each sample using coefficients for each gene in the RT-qPCR signature that were calculated by a generalized logistic regression model (GLM) contrasting MIS-C to KD, DV, and DB (details in Supplementary Methods). The performance of the RT-qPCR signature was evaluated through the weighted DRS in ROC models that contrasted MIS-C to previously “seen” groups included in the discovery set—KD, DV, and DB—and then separately with COVID-19 which was not included in the discovery cohort.

### Data Availability

Gene counts and patient metadata from EUCLIDS RNA-Seq (including KD patients) are available at ArrayExpress under accession E-MTAB-11671. The merged and normalized dataset used for the analysis of the discovery dataset is available in ArrayExpress (E-MTAB-12793). Links to raw data files can be found at the above accessions on the European Bioinformatics Institute platform. The code used in the analyses can be found at: https://github.com/PIDBG/misc_transcriptomic_signature.

## RESULTS

**Figure 1.**
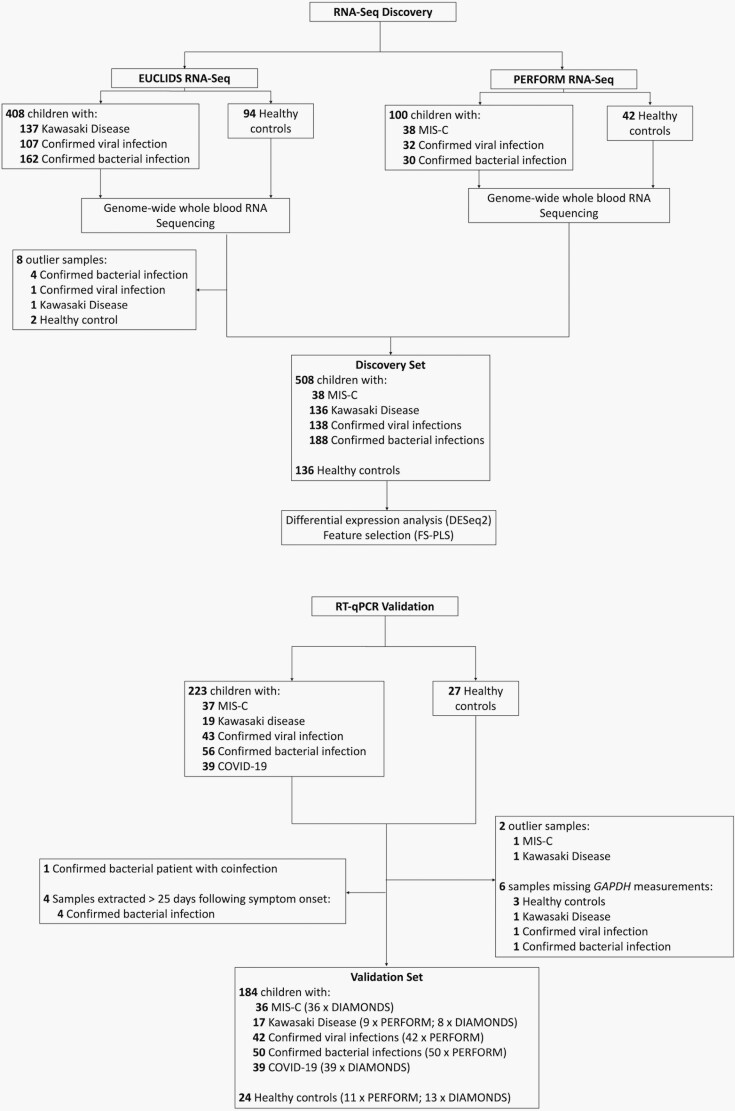
Consort diagram showing the cohorts included in this study and the key steps performed. Outliers were determined using the Hotelling’s *t*-squared test with a confidence level of 0.99 using values from principal components (PC) 1 and 2 from principal component analysis (PCA).

To identify an RNA signature for diagnosing MIS-C, we established a discovery set and a separate validation set (Figure 1). Whole-blood transcriptomes of MIS-C patients (*n =* 38) and patients with KD (*n =* 136), viral infections (*n =* 138), bacterial infections (*n =* 188), and healthy controls (*n =* 134) were included in the discovery cohort.

MIS-C patients were older than the comparator disease groups (median age 126 months vs. 30 months for KD, 7 months for viral, and 38 months for bacterial) and had a longer median duration of symptoms prior to presentation (6 days) than the bacterial infection groups (2 days for bacterial), but similar to KD (6 days) and viral infections (5 days) (Table 1). A high proportion of MIS-C patients presented with shock (52%, *n =* 37), 60.6% (*n =* 23) were admitted to intensive care, 52% (*n =* 20) required inotropes, and 13.2% (*n =* 5) and 10.5% (*n =* 4) required noninvasive and invasive ventilation, respectively. The causative pathogens for the confirmed bacterial and viral infections are summarized in Supplementary Table 1.

**Table 1.** Demographic and Clinical Characteristics of the Patients With MIS-C, KD, DB, and DV Infections Used in the Case–Control Discovery Set and the RT-qPCR Validation Set. Values Are Median (IQR) Unless Stated

	Discovery				Validation				
	MIS-C (*n* = 38)	KD (*n* = 136)	Viral (*n* = 138)	Bacterial (*n* = 188)	MIS-C (*n* = 36)	KD (*n* = 17)	Viral (*n* = 42)	Bacterial (*n* = 50)	COVID-19 (*n* = 39)
Age in months	126 (65–151)	30 (16–52)	7 (2–20)	38 (11–96)	103 (64–145)	26 (15–46)	42 (18–112)	46 (7–114)	16 (2–162)
Sex at birth (female, %)	13 (39%)	59 (43%)	45 (33%)	85 (45%)	12 (33.3%)	7 (41%)	16 (38%)	25 (50%)	14 (36%)
Days since symptom onset	6 (5–7)	6 (5–8)	5 (2–6)	2 (1–4)	5.5 (4–7)	5 (4–8)	4 (2–6)	2 (1–4.5)	2 (1–5)
Required inotropic support (*n*, %)	20 (52.6%)	2 (1.4%)	3 (2%)	50 (26.6%)	15 (42%)	0	1 (2.4%)	5 (10%)	1 (2.6%)
Required noninvasive ventilation (*n*, %)	5 (13.2%)	2 (1.4%)	6 (4.3%)	46 (24%)	2 (6%)	1 (6%)	1 (2.4%)	3 (6%)	2 (5%)
Required invasive ventilation (*n*, %)	4 (10.5%)	0	7 (5.1%)	16 (8.5%)	3 (8%)	0	2 (5%)	12 (24%)	0
Admitted to PICU (*n*, %)	23 (60.6%)	1 (0.7%)	25 (18%)	84 (44.7%)	16 (44%)	1 (6%)	4 (10%)	18 (36%)	2 (5%)
White blood cells (109/L)	9.7 (8.1–11.5)	13.2 (10.4–18)	9.9 (6–14)	13.2 (8.1–20.6)	8.2 (6.5–12.3)	12.3 (8.9–13.9)	10.1 (6.5–13.2)	12.5 (8.1–21.6)	7.9 (5.6–9.3)
Neutrophils (109/L)	8.1 (4.5–11.0)	9.2 (6.3–11.5)	3.6 (1.8–6.5)	14.1 (6–47)	6.5 (4.5–8.7)	6.7 (4.9–11.6)	5.1 (3.4–7.6)	7.0 (3.4–15.2)	3 (1.9–4.4)
Lymphocytes (109/L)	1.2 (0.8–1.8)	2.8 (1.7–4.9)	4.7 (2.8–6.7)	2.8 (1.5–8.6)	1.1 (0.6–1.6)	2.4 (1.7–5.2)	2.6 (1.7–4.9)	1.9 (0.9–2.9)	2.8 (1.6–4.9)
Monocytes (109/L)	0.3 (0.2–0.4)	0.7 (0.5–1.1)	0.8 (0.5–1.4)	1.6 (0.7–4.8)	0.2 (0.2–0.4)	0.7 (0.5–1)	0.9 (0.5–1.3)	0.9 (0.5–1.3)	0.7 (0.5–1.2)
Platelets (109/L)	209 (144–300.5)	343 (257–426)	365 (245–509)	243 (157–329)	169 (124–251)	382 (279–433.5)	261 (194–326)	289 (235–332)	294 (230–355)
CRP (mg/L)	173 (99–250)	73 (43–162)	13.2 (4.1–38)	116.8 (40.3–212)	193 (80–230)	51 (42–146)	5.9 (2.9–22)	57 (12.5–129.4)	3 (1–7.5)

Abbreviation: IQR, interquartile range; KD, Kawasaki disease; MIS-C, multisystem inflammatory syndrome in children.

The patients in the validation cohort were similar to those in the discovery set, apart from a different range of causative pathogens (Table 1 and Supplementary Table 1). The validation cohort was composed of patients with MIS-C (*n =* 37), KD (*n* = 17), confirmed viral infections not including COVID-19 (*n =* 41), COVID-19 (*n =* 39), confirmed bacterial infections (*n =* 50), and healthy controls (*n =* 24) (Supplementary Methods). See Figure 1 for details of samples that were excluded. Principal component (PC) analysis showed visible clustering of patients according to disease group (Supplementary Figure 1A), with strong correlations between PC 1 and each of the disease groups (Supplementary Figure 2).

### RNA Differential Expression Analysis

Overall, 5696 genes were found to be SDE (BH-adjusted *P* value < .05) between MIS-C and the combined KD, viral and bacterial infection groups with 3250 and 2446 genes over- and underexpressed in MIS-C, respectively (Supplementary Figure 3A and Supplementary File 1). For MIS-C versus KD, 4786 genes were SDE (2681 and 2105 genes over- and underexpressed respectively; Supplementary Figure 3B and Supplementary File 2). For MIS-C versus viral infection, 10 654 genes were SDE (5973 and 4681 genes over- and under-expressed respectively; Supplementary Figure 3C and Supplementary File 3). For MIS-C versus bacterial infection, 3718 genes were SDE (1776 and 1942 genes over- and under-expressed respectively; Supplementary Figure 3D and Supplementary File 4). T-Cell Receptor Beta Variable 11-2 (*TRBV11-2*) was the top SDE gene for MIS-C versus the comparator groups combined (BH-adjusted *P* value: 7.144 × 10^–27^; LFC: 1.99) (Supplementary Figure 3A).

### Identification of Diagnostic Combination of Genes

**Figure 2.**
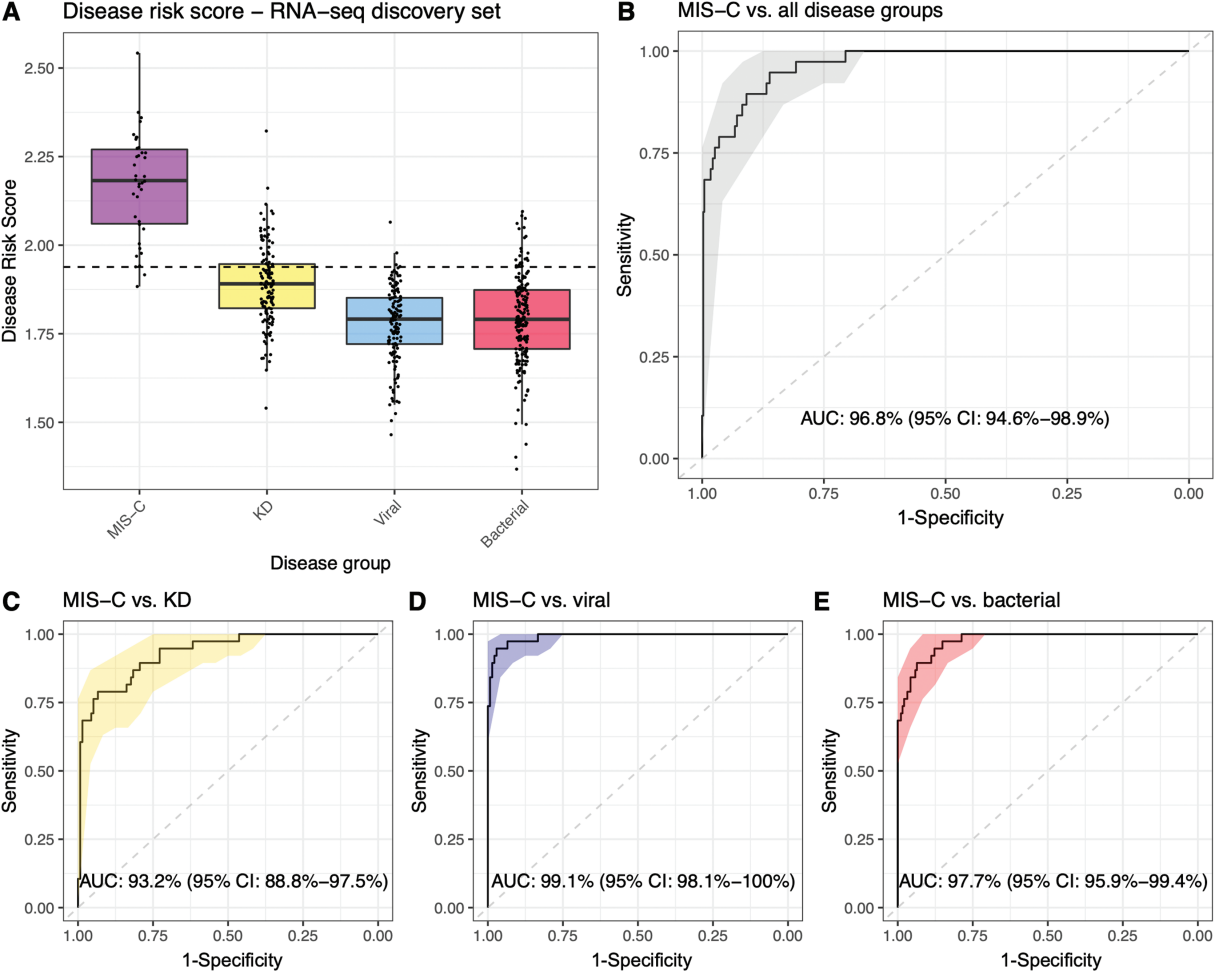
Performance of the 5-gene combination in the RNA-Seq discovery set. (**A**) Boxplots showing the disease risk score for patients from the different disease groups included in the RNA-Seq discovery set. The dashed line represents the threshold of classification calculated using the Youden’s index by maximizing both sensitivity and specificity of MIS-C versus KD, viral and bacterial. (**B**) ROC curve for the performance of the combination of 5 genes identified in the discovery dataset in the MIS-C versus KD, viral, and bacterial combined. (**C**) ROC curve for the performance of the signature for MIS-C. versus KD. (**D**) ROC curve for the performance of the signature for MIS-C versus viral; and (**E**) ROC curve for the performance of the signature for MIS-C versus bacterial. AUCs and 95% confidence intervals (CIs) are printed on the plots. KD: Kawasaki disease; MIS-C: multisystem inflammatory syndrome in children.

Variable selection using FS-PLS identified 4 genes distinguishing MIS-C from KD, viral and bacterial infections: HSPB1 Associated Protein 1 (*HSPBAP1*), Vacuolar Protein Sorting-Associated Protein 37C (*VPS37C*), Transforming Growth Factor Beta 1 (*TGFB1*) and MX Dynamin Like GTPase 2 (*MX2*). To introduce redundancy to the gene set for subsequent transfer to RT-qPCR assays, the top SDE gene—*TRBV11-2*—was also included. *TRBV11-2* was not selected by FS-PLS due to cutoffs introduced to avoid the risk of overfitting (Supplementary Methods). When the 5 genes were combined as a weighted DRS for each patient (Figure 2A), MIS-C was distinguished from the combined comparator diseases with AUC of 96.8% (95% CI: 94.6%–98.9%; Figure 2B) with a sensitivity of 86.1% and specificity of 94.7% (Figure 2A). When the individual comparator groups were considered separately, the 5-gene combination achieved an AUC of 93.2% (95% CI: 88.8%–97.5%; Figure 2C) for MIS-C versus KD, 99.1% (95% CI: 98.1%–100%; Figure 2D) for MIS-C versus viral, and 97.7% (95% CI: 95.9%–99.4%; Figure 2E), for MIS-C versus bacterial.

### RT-qPCR Validation

The 5 candidate biomarker genes identified from the discovery set were taken forward to RT-qPCR validation together with the “housekeeping” gene *GAPDH*. The 5 candidate genes were combined into a gene signature—the RT-qPCR signature—and the performance of the signature was evaluated in the validation set.

**Figure 3.**
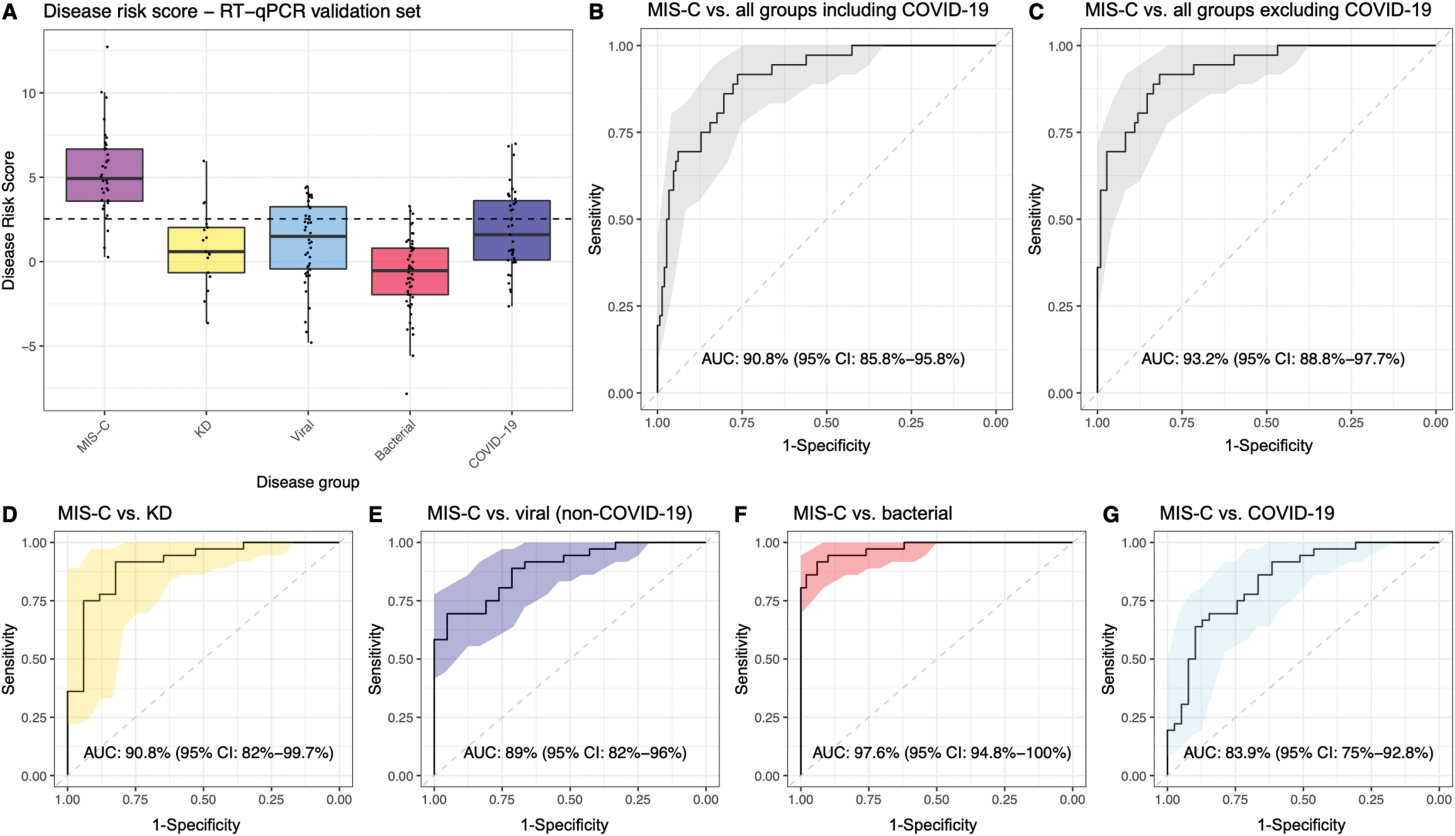
Performance of the 5-gene RT-qPCR signature in the RT-qPCR validation set. (**A**) Boxplots showing the disease risk score for patients from the different disease groups. The dashed line represents the threshold of classification for the validation set calculated using the Youden’s index by maximizing both sensitivity and specificity of MIS-C versus KD, viral and bacterial. Sensitivity = 91.7% and specificity = 81.7% for MIS-C versus KD, viral and bacterial. **B:** ROC curve for evaluating the performance of the 5-gene RT-qPCR signature in contrasting MIS-C from all comparator groups including COVID-19. (**C**) ROC curve showing performance for MIS-C versus all comparator groups excluding COVID-19. (**D–G**) performance in contrasting MIS-C from individual comparator groups. AUCs and 95% confidence intervals (CIs) are printed on the plots. KD, Kawasaki disease; MIS-C, multisystem inflammatory syndrome in children; DB, combined sterile site and nonsterile site bacterial groups.

To optimize the performance of the signature on a different platform, a weighted DRS for each patient was calculated (details in Supplementary Methods, with gene coefficients in Supplementary Results). ROC plots and AUCs were calculated using the weighted DRS for all patients to evaluate the signature’s performance at distinguishing MIS-C and comparator phenotypic groups either with or without the COVID-19 group which was not included in the discovery set. The 5-gene RT-qPCR signature achieved a sensitivity of 91.7% and specificity of 81.7% for distinguishing MIS-C from the disease groups included in the discovery set—excluding COVID-19—with an AUC of 93.2 (95% CI: 88.8%–97.7%; Figure 3A and C).

When the signature was assessed in the specific disease groups, the highest AUCs were observed when distinguishing MIS-C from bacterial infections with an AUC of 97.6% (95% CI: 94.8%–100%; Figure 3F). When bacterial patients were split into the sterile sites and nonsterile site bacteria, the 5-gene RT-qPCR signature achieved an AUC of 97.8% (95% CI: 95.3%–100%, data not shown) for MIS-C versus sterile site bacteria and 96% (95% CI: 90.3%–100%, data not shown) for MIS-C versus nonsterile site bacteria. The 5-gene RT-qPCR signature achieved an AUC of 90.8% (95% CI: 82%–99.7%) for distinguishing between MIS-C versus KD (Figure 3D), 89% (95% CI: 82%–96%) for MIS-C versus viral (Figure 3E), and 83.9% (95% CI: 75%–92.8%) for MIS-C versus COVID-19 (Figure 3G). The 5-gene RT-qPCR signature achieved an AUC of 90.8 (95% CI: 85.8%–95.8%) when contrasting MIS-C against all phenotypic groups including COVID-19 (Figure 3B). The performance of the 5-gene signature was evaluated in various subgroup analyses to confirm that its distinguishing power was not driven by any potentially confounding factors (Supplementary Results). From these analyses, it is evident that the performance of the 5-gene signature is not driven by differences in whether patients received inotropic support, the duration of their symptoms at the time of sampling, or their age.

## DISCUSSION

We used whole-blood RNA-Seq to compare the blood transcriptomes of patients with MIS-C to those with KD, bacterial, and viral infections. Despite overlapping clinical and laboratory features between these conditions, there were clear differences in the blood transcriptome, with 4786, 10 654 and 3718 SDE genes between MIS-C versus KD, versus viral, and versus bacterial infections, respectively, providing further support that MIS-C is a biologically distinct entity.

Using feature selection tools, we identified a minimal gene signature distinguishing MIS-C from the other disease groups. We performed cross-platform validation of the biomarker genes in an independent patient set using RT-qPCR, a methodology more suited for clinical use of the signature as a diagnostic test. Patients included reflected not only disease groups in the discovery set (MIS-C, KD, viral, and bacterial infections) but also COVID-19. Cross-platform validation of the genes identified by RNA-Seq to RT-qPCR in the independent patient cohort showed a similar performance of the RT-qPCR signature to that in the RNA-Seq discovery set.

Since the emergence of MIS-C in 2020, diagnosis has been challenging, as the clinical and laboratory features are often indistinguishable from those of a wide range of other diseases. There has been particular difficulty in distinguishing MIS-C from KD, with reports early in the pandemic from several countries documenting an upsurge in KD-like inflammatory syndromes [7, 34], which with hindsight may have been misdiagnosed of MIS-C as KD. In addition, similarities between MIS-C and sepsis, septic shock, staphylococcal, and streptococcal shock syndromes present considerable difficulties in terms of managing patients [3, 12, 35], as administering immunomodulating agents (the usual treatment for MIS-C) to patients with severe infections may be deleterious. Additionally, many patients who turn out to have MIS-C receive prolonged courses of broad-spectrum antibiotics before the diagnosis is made. The diagnostic difficulty is most acute in low- and middle-income countries, where a wide range of prevalent infections share features with MIS-C.

The overlap in clinical features between MIS-C and KD led to immunoglobulin—the proven treatment for KD—being widely adopted as the initial treatment for MIS-C. As there is evidence that MIS-C outcome may be no different when steroids are used instead of IVIG [15], and since immunoglobulin is expensive with limited availability in some countries, a test capable of distinguishing between the 2 disorders would be helpful in targeting specific treatments for each disease.

Although we have focused on the use of genes as diagnostic biomarkers, the extensive RNA-Seq data comparing MIS-C with other diseases will provide the scientific community with a valuable resource for exploring the biological pathways and host response distinguishing MIS-C from other disorders. Further biological exploration of the data is likely to identify novel mechanisms and pathways mediating MIS-C as targets for novel therapies.

The genes included in the 5-gene RT-qPCR signature have biological functions that may provide insight into the pathogenesis of MIS-C. *HSPBAP1* regulates the expression of *TMPRSS2*, the gene encoding the enzyme transmembrane protease, serine 2, which is used by SARS-CoV-2 for S protein priming [36]. *TGFB1* encodes a ligand that, among other functions, modulates the expression and activation of cytokines including interferon-gamma and tumor necrosis factor-alpha (TNF-α). Elevated expression of *TGFB1* in CD4^+^ T cells and higher numbers of TGFB1-expressing T and CD14-positive cells have been observed in a small set of patients with severe COVID-19 [37]. *VPS37C* is involved in endosomal sorting and has been shown to play a role in viral budding, specifically of human immunodeficiency virus, type 1 (HIV-1) by the HIV-1 Gag protein [38]. *MX2* encodes a GTPase and is part of the antiviral response induced by type 1 and 3 interferons. Elevated levels of *MX2* have been identified in individuals with SARS-CoV-2 [39], and other viral infections [40]. Our finding that *TRBV11-2* is the most SDE gene is in keeping with previous reports, where an expansion of peripheral T cells carrying *TRBV11-2* has been correlated with disease severity and serum cytokine levels [41, 42]. Furthermore, it has been postulated that the selective expansion of specific T-cell V beta subsets is being driven by a super-antigen mediated process [41–44].

Our study has several limitations. The MIS-C patients included in the signature discovery and validation sets were recruited during the period of April 2020–April 2021. As such, the SARS-CoV-2 variants that triggered the illness are limited to those that were circulating during this period and notably, would not include the Omicron variant that was first detected in November 2021. The Omicron variant appears to be linked with less severe manifestations of MIS-C [45–47]. External validation of the gene panel in larger numbers of patients across different periods of the pandemic, and different global settings, will be needed to assess the robustness of the signature in the face of different viral pathogen genetics. A limitation of all studies exploring patients with MIS-C and KD is that there is no gold-standard diagnostic test for either illness. However, the KD patients included in the discovery set were recruited prior to the COVID-19 pandemic. For MIS-C, we made every effort to only include in both the discovery and validation patients meeting the WHO definition, including evidence of recent SARS-CoV-2 infection detected by antibody or PCR.

The patients presented here were recruited into sequential studies (EUCLIDS, PERFORM, and DIAMONDS). Some phenotypic groups are composed of patients from only 1 study, such as MIS-C and COVID-19 who were recruited into DIAMONDS. Despite the almost identical clinical recruitment and laboratory protocols, as well as phenotyping algorithms, the study into which patients were recruited may affect the expression levels of genes included in the 5-gene signature. Despite these limitations, our data provide evidence that MIS-C is a distinct syndrome characterized by a unique transcriptomic signature, and that a diagnostic test based on a small number of RNA transcripts is achievable.

Our finding that MIS-C can be distinguished from other conditions using a small RNA signature, and that detection of this signature by RT-qPCR achieve similar accuracy to that of RNA-Seq, paves the way for the development of a rapid test to distinguish MIS-C from other phenotypically similar diseases. As a range of devices that can provide RT-qPCR results rapidly are now available, including several platforms already in use in clinical settings worldwide [48–50], the development of a clinically applicable test for MIS-C based on the 5-gene RT-qPCR signature can be readily achieved.

## Supplementary Material

piad035_suppl_Supplementary_Material

## Notes

***Financial support.*** This work was supported by the European Union’s Horizon 2020 Program under grants (848196 DIAMONDS, 668303 PERFORM, 279185 EUCLIDS, Prof Levin), by the Imperial Biomedical Research Centre (BRC) of the National Institute for Health Research (NIHR), grants (206508/Z/17/Z and MRF-160-0008-ELP-KAFO-C0801 to Dr Kaforou) from the Wellcome Trust and the Medical Research Foundation, a grant (215214/Z/19/Z to Dr Jackson) from the Wellcome Trust, a grant (R61HD105590-01 PreVAIL kIds to Dr. Burns) from the National Institutes of Health, and grants (WDPI_G28062 and WDPI_P89720 to Drs Herberg and Georgiou) from the Community Jameel Imperial College COVID-19 Excellence Fund and the Rosetrees Trust. This work has been supported by the Imperial Confidence in Concept Scheme, funded by MRC Confidence in Concept, Wellcome Trust Institutional Strategic Support Fund, NIHR Imperial BRC, and Rosetrees Trust (to Rodriguez-Manzano and Kaforou).

***Potential conflicts of interest.*** The authors have filed IP on the 5-gene signature described in this study. The authors have no other conflicts of interest to declare. The funders had no role in the study design, the collection, analysis, and interpretation of data, the writing of the report, and the decision to submit the manuscript for publication. Heather Jackson wrote the first draft of the manuscript. No honorarium, grant, or other form of payment was given to anyone to produce the manuscript.

***Data Availability.*** Gene counts and patient metadata from EUCLIDS RNA-Seq (including KD patients) are available at ArrayExpress under accession E-MTAB-11671. The merged and normalized dataset used for the analysis of the discovery dataset is available in ArrayExpress (E-MTAB-12793). The code used in the analyses can be found at: https://github.com/PIDBG/misc_transcriptomic_signature.
