# Supplementary material for: Diagnosis of Multisystem Inflammatory Syndrome in Children by a Whole-Blood Transcriptional Signature
Source: J Pediatric Infect Dis Soc. 2023 May 31;12(6):322–31. doi: 10.1093/jpids/piad035 (PMC10312302; doi:10.1093/jpids/piad035)
Supplement: piad035_suppl_Supplementary_Material [file piad035_suppl_supplementary_material.docx]

**Table of Contents**

[1. Legend for Supplementary Files 2](#_Toc126767372)

[2. Supplementary Methods 2](#_Toc126767373)

[2.1. Patients in the Discovery and Validation Sets 2](#_Toc126767374)

[2.2. RNA isolation and quantification 3](#_Toc126767375)

[2.3. Discovery of a diagnostic gene signature 4](#_Toc126767376)

[**2.3.1.** **Data pre-processing and normalisation** 4](#_Toc126767377)

[**2.3.2.** **Signature identification with FS-PLS and signature evaluation** 4](#_Toc126767378)

[2.4. RT-qPCR gene quantification 5](#_Toc126767379)

[**2.4.1.** **RT-qPCR data analysis** 6](#_Toc126767380)

[3. Supplementary Results 7](#_Toc126767381)

[3.1. Healthy controls 7](#_Toc126767382)

[3.2. Principal Component Analysis 7](#_Toc126767383)

[3.3. RT-qPCR sample exclusions and data analysis 8](#_Toc126767384)

[3.4. Sub-group analyses exploring the effects of confounding on the 5-gene signature 8](#_Toc126767385)

[3.5. Sub-analyses contrasting MIS-C to Kawasaki disease 13](#_Toc126767386)

[3.6. Retraining the 5-gene RT-qPCR validation signature weights with COVID-19 11](#_Toc126767387)

[4. Supplementary Figures 15](#_Toc126767388)

[5. Supplementary Tables 18](#_Toc126767389)

[6. DIAMONDS, PERFORM and EUCLIDS Consortia 20](#_Toc126767391)

[6.1. DIAMONDS Consortium 20](#_Toc126767392)

[6.2. PERFORM Consortium 34](#_Toc126767393)

[6.3. EUCLIDS Consortium 45](#_Toc126767394)

# **Legend for Supplementary Files**

Supplementary File 1 – genes significantly differentially expressed (SDE) between MIS-C *vs*. KD, viral and bacterial infections.

Supplementary File 2 – genes significantly differentially expressed (SDE) between MIS-C *vs*. KD.

Supplementary File 3 – genes significantly differentially expressed (SDE) between MIS-C *vs*. viral infections.

Supplementary File 4 – genes significantly differentially expressed (SDE) between MIS-C *vs*. bacterial infections.

# **Supplementary Methods**

# **Patients in the Discovery and Validation Sets**

The clinical studies from which the patients included in the RNA transcriptomic studies were selected were successive studies of children and adults with infectious and inflammatory diseases. EUCLIDS (European Union Childhood Life-Threatening Infectious Disease Study) recruited patients from 9 EU countries 2011-2016, PERFORM (Personalised Risk assessment in Febrile illness to Optimise Real-life Management across the European Union) recruited children from 10 EU countries (2016-2020, and DIAMONDS (Diagnosis and Management of Febrile Illness using RNA Personalised Molecular Signature Diagnosis) commenced recruitment in 2020, and is ongoing. In addition, children with KD were also recruited in the United States through the Kawasaki Disease Research Center at the University of California San Diego (UCSD). In each of these studies, children with suspected inflammatory or infectious disorders were recruited and phenotyped according to a standardised algorithm. Patients were recruited at the earliest time point following presentation to hospital, and blood samples for research were taken at the same time as routine clinical investigations where possible. The majority of patients were recruited in hospital accident and emergency departments, or on transfer to wards or intensive care units. Patients were assigned to final phenotype groups after review of all clinical, laboratory and imaging results, and after review by at least two experienced clinicians. The phenotyping algorithm has been described previously^1,2^.

The discovery set was composed of two batches named after the study from which the majority of patients originated (EUCLIDS RNA-Seq and PERFORM RNA-Seq). In the discovery set DB, DV and HC patients originated from the EUCLIDS or PERFORM studies, whilst in the validation set these groups originated from only the PERFORM study. MIS-C and COVID-19 patients always originated from the DIAMONDS study. KD patients originated from the UCSD study in the discovery set and PERFORM or DIAMONDS in the validation set (Figure 1). There was no overlap between the patients included in the discovery set and the validation set.

**Case Definitions:**

**MIS-C** classification was based on patients meeting the full WHO MIS-C case definition ^3^:

Children and adolescents 0–19 years of age with fever > 3 days

**AND** two of the following:

1. Rash or bilateral non-purulent conjunctivitis or muco-cutaneous inflammation signs (oral, hands or feet).
2. Hypotension or shock.
3. Features of myocardial dysfunction, pericarditis, valvulitis, or coronary abnormalities (including ECHO findings or elevated Troponin/NT-proBNP),
4. Evidence of coagulopathy (by PT, PTT, elevated d-Dimers).
5. Acute gastrointestinal problems (diarrhoea, vomiting, or abdominal pain).

**AND**

Elevated markers of inflammation such as ESR, C-reactive protein, or procalcitonin.

**AND**

No other obvious microbial cause of inflammation, including bacterial sepsis, staphylococcal or streptococcal shock syndromes.

**AND**

Evidence of COVID-19 **(**RT-PCR, antigen test or serology positive), or likely contact with patients with COVID-19.

**Confirmed Bacterial Infection – Definite Bacterial (DB):** The DB group included patients in whom an appropriate bacterial pathogen was isolated from a normally sterile site, and clinical presentation is consistent with the pathogen isolated. In addition, pathogens normally only detected on mucosal surfaces or diagnosed serologically (*M. tuberculosis*, *B. pertussis*, *M. pneumoniae*, *Borrelia* species, *Campylobacter* and *Salmonella)* were included We termed these non-sterile site definite bacterial infections (NSDB). As MIS-C frequently presents with gastrointestinal symptoms, resembling enteric infections, or with rash, neurological and multisystem features that can be seen in Lyme disease and mycoplasma infections, we considered the wider inclusion group as an important comparator group for this study.

**Confirmed Viral Infection – Definite Viral (DV):** The DV group required identification of virus matching syndrome in addition to C-reactive protein (CRP) levels ≤ 60 mg/L.

**Kawasaki Disease** **(KD):** diagnosis was based on the American Heart Association diagnostic criteria ^4^.

**COVID-19** was diagnosed in children presenting to hospital with febrile or respiratory illness, and PCR confirmation of SARS-CoV-2 in respiratory secretions, and with no other potential cause of the illness identified

**Healthy controls:** Otherwise healthy children attending hospitals for non-infectious or inflammatory conditions or for pre surgery screening were recruited to EUCLIDS, PERFORM or DIAMONDS. These control children were used in the RNA sequencing studies solely for normalisation of sequencing runs.

## **RNA isolation and quantification**

RNA extraction: Blood samples for transcriptional studies were collected in PAXgene™ Blood RNA vacutainers (Pre-Analytix (Applied Biosystems) and stored at –80^o^C. For each study, all samples were shipped frozen and extracted after randomisation across patient diagnostic categories to reduce experimental variation using recommended isolation kits. Total RNA (including miRNA) was isolated and underwent additional DNAse treatment (Turbo DNAse (Ambion) or RNA Clean & Concentrator™ (Zymo Research)). Samples were assessed using Nanodrop™ spectrophotometer (Thermo Fisher Scientific), QIAxcel (Qiagen), Bioanalyser (Agilent) and Qubit fluorometer (Invitrogen) before normalisation and plating for sequencing.

RNA Sequencing: Genomic material was quantified using RiboGreen (Invitrogen) on the FLUOstar OPTIMA plate reader (BMG Labtech) and the size profile and integrity analysed on the 2200 TapeStation (Agilent, RNA ScreenTape). Input material was normalised, and strand specific library preparation was completed using NEBNext® Ultra™ II mRNA kit (NEB) and NEB rRNA/globin depletion probes following manufacturer’s instructions. Libraries were prepared on a Tetrad (Bio-Rad) using in-house unique dual indexing primers (based on ^5^). Individual libraries were normalised using Qubit and pooled together. The pooled library was diluted to ~10 nM for storage and denatured and further diluted prior to loading on the sequencer.

## **Discovery of a diagnostic gene signature**

### **Data pre-processing and normalisation**

Whole blood transcriptomes used in this study originated from two RNA-Seq studies (EUCLIDS and PERFORM). From the EUCLIDS study, there were samples from 136 KD patients, 158 DB and 106 DV patients and 92 healthy controls. From the PERFORM study, there were samples from 38 patients with MIS-C, 30 with DB infections, 32 with DV infections, and 42 healthy controls. The healthy controls were used for normalisation and merging of the two sequencing studies to avoid any batch effects but were not used in the discovery of RNA signatures. Prior to merging of the two datasets, pre-processing was performed on each dataset separately. Ribosomal genes, genes in the depletion kit *(HBB, MT-RNR2, MT-RNR1, HBA2, HBG2, HBG1, HBA1)*, and genes without counts ≥10 in at least three samples were removed from the separate datasets. Principal component analysis (PCA) was performed with outliers identified and removed. Outliers were determined using the Hotelling’s t-squared test with a confidence level of 0.99. Plate and site effects and effects of sampling tube (PAXgene tubes were used for the majority but a small proportion of patients in the EUCLIDS study had samples collected into Tempus tubes) were removed using combat_seq from the sva package ^6^. The counts from the two datasets were merged using only genes present in both datasets. Combat_seq was used to correct for the experimental technical effects between the two studies and counts were normalised using DESeq2 ^7^. Genes without counts ≥20 in at least three samples were removed from the normalised dataset.

### **Signature identification with FS-PLS and signature evaluation**

The previously reported feature selection method FS-PLS (Forward Selection Partial Least Squares) has been used for identification of sparse biomarker signatures, as it excludes genes that show a high degree of correlation ^8^. This approach was modified to enable simultaneous comparison of multiple disease groups. FS-PLS was run with the following comparisons included: MIS-C *vs*. KD+DV+DB (both RNA-Seq datasets); MIS-C *vs.* KD+DV+DB (EUCLIDS RNA-Seq only); MIS-C *vs.* DV+DB (PERFORM RNA-Seq only; no KD samples present in PERFORM RNA-Seq), MIS-C *vs*. DB; MIS-C *vs*. DV; and MIS-C *vs*. KD. FS-PLS was run using genes with adjusted *p*-values <0.001 and absolute LFC >0.5 in at least one of MIS-C *vs*. KD, *vs*. DV, or *vs.* DB, and with mean counts >50 in all disease groups and >100 reads in at least one disease group.

The top significantly differentially expressed (SDE) gene for MIS-C *vs.* KD+DV+DB was added into the signature identified by FS-PLS to improve chances of translation across platforms and to introduce redundancy into the signature.

The performance of the combination of the FS-PLS signature and the top SDE gene was evaluated through calculating AUCs using a score for each patient – the weighted disease risk score (DRS). The weighted DRS is an adaptation of the DRS utilised and described in ^8-10^. In brief, normalised gene counts were multiplied by the gene coefficients calculated from a generalised logistic regression model (GLM) contrasting MIS-C to all other samples (KD, DV, DB). The weighted DRS was calculated per sample (*i*) as follows:

$$Weighted Disease Risk {Score}^{i}= \sum_{l=0}^{m} normalised gene {counts}_{m}^{i}\times{gene coefficients}_{m}^{i}$$

Where:

**i** represents each sample;

**m** represents each gene included in the signature.

## **RT-qPCR gene quantification**

For the development of the RT-qPCR assays, exon counts corresponding to the genes included in the 5-gene signature were extracted from the exon counts quantified from the raw sequencing data through using featureCounts ^11^ followed by DEXSeq ^12^. This was to determine the optimal gene region to use in primer sequences. Only exon counts from PERFORM study were used since this study included MIS-C patients. For each gene, the optimal exon for validation was identified, taking into account the AUC and log fold-change values for MIS-C vs DB+DV, in addition to the mean exon counts overall and within each disease group.

Primers were designed for the genes included in the diagnostic signature. Gene sequences were downloaded from Ensembl Genome Browser (<https://www.ensembl.org/index.html>) using as reference human genome the assembly GRCh38.p13. Primer design and *in-silico* analysis were conducted using GENEious Prime 2022.0.1 (<https://www.geneious.com>). Primer characteristics were evaluated through IDT OligoAnalyzer software (<https://eu.idtdna.com/pages/tools/oligoanalyzer>) using the J. SantaLucia thermodynamic table for melting temperature (T_m_) calculation, hairpin, self-dimer, and cross-primer formation (<https://www.thermofisher.com/uk/en/home/brands/thermo-scientific/molecular-biology/molecular-biology-learning-center/molecular-biology-resource-library/thermo-scientific-web-tools/multiple-primer-analyzer.html>). Intercalating dye-based PCR assays were designed and developed and purchased from IDT Integrated DNA Technologies (IDT, Coralville, IA).

We assessed the performance of the signature using a Biomark HD high-throughput qPCR platform and 192.24 Dynamic Array integrated fluidic circuit (IFC) following manufacturer instructions. The experimental workflow consisted of 4 steps: reverse transcription (RT), pre-amplification, on-chip gene expression, and data analysis.

*Reverse transcription*

The first step consisted of performing RT using the Fluidigm Master Mix, which contains all components required for cDNA synthesis, including buffer dNTPs, ribonuclease inhibitor and an engineered RNA reverse transcriptase (Fluidigm product numbers: #100-5580 and #100-5581). Each RT reaction was performed in 5 μL of final reaction volume with 1 μL of Reverse Transcription Master Mix, 3 μL of RNA- free water and 1 μL of RNA (2.5 pg/μL - 250 ng/μL). The reaction was incubated for 5 minutes at 25°C, 30 minutes at 42°C and 5 minutes at 85°C. This was performed in a conventional real- time qPCR instrument (Roche LightCycler96).

*Pre-amplification (pre-amp)*

In a microcentrifuge tube, we combined an equal volume of each of the five RT-PCR assays together with the GAPDH assay from initial concentration of 5 µM for each primer. The pool was diluted in a buffer, containing 10 mM Tris, pH 8.0, 0.1 mM EDTA, to reach a final concentration of 0.2X (100 nM). The pre-amp reaction was performed in a total volume of 5 μL, which includes 1 μL of Pre-Amp master mix (Fluidigm), 1.25 μL of pooled TaqMan assay mix (0.2X), 1.5 μL of nuclease-free water and 1.25 μL of cDNA for 2 minutes, followed by 20 cycles of 95°C for 15 seconds and 60°C for 4 minutes. This was performed in a conventional real-time qPCR instrument (Roche LightCycler96).

*On-chip gene expression*

Each pre-amplified sample was added to a pre-mix solution as follows: 2 μL EvaGreen-fluorescence dyes master mix with Low Rox (Bio-Rad), 0.2 μL of 20X GE Sample Loading Reagent (Fluidigm) and 1.8 μL of cDNA pre-amplified in a final volume of 4 μL. All the reagents, samples (3 μL each sample) and assays (3 μL of each primer set at 500 nM) were added to the chip before placing it into the RX controller to prime and load. The 192.24 IFC was then placed in the Biomark HD using the GE 192×24 Standard v1.pcl cycling program (Fluidigm) according to the recommended settings given in the manufacturer's 192.24 Fast/Standard Gene Expression Workflow protocol. Each experimental condition was conducted in quadruplicates.

### **RT-qPCR data analysis**

The real-time data from RT-qPCR was pre-processed by performing baseline correction and cycle thresholds (CT) values were extracted using a global threshold, as determined by Fluidigm’s Real-Time qPCR Software (version 4.1.2).

CT values of 40 were treated as missing. Samples missing measurements for ≥3 of the 4 *GAPDH* assay replicates were excluded. For each sample, the standard deviation for each gene across replicates was calculated. For genes with a standard deviation ≤3 across replicates, the mean CT value was taken for a given sample. For genes with standard deviations across replicates >3, CT values were manually examined and either set to NA or the mean across fewer replicates was taken (i.e., 2 replicates) if those replicates had a standard deviation ≤3. For each sample, for each gene, the averaged *GAPDH* values were subtracted from the averaged gene values to obtain normalised gene measurements.

Weighted DRS were calculated for each sample as shown in the equation in 2.3.2. Gene coefficients were calculated by performing a GLM that contrasted MIS-C to KD, DV and DB combined. An additional model was also used which contrasted MIS-C to KD, DV, DB and COVID-19. Through using the caret R package ^13^, 10-fold cross validation was implemented in the GLM to calculate cross-validated gene coefficients which were used for the weighted DRS calculation. The weighted DRS were used for ROC analysis and for calculating AUCs for MIS-C *vs*. all comparator groups (KD, DV, DB, COVID-19) with and without COVID-19 (since COVID-19 was not used in signature discovery).

Stratified analyses were also performed to verify that the observed performance of the gene signature was not driven by possibly confounding factors, including age, requirement of inotropes and duration of illness at time of sampling. Specific analyses were performed for the MIS-C *vs*. KD comparison, including stratifying patients according to whether they required PICU and based on age. Furthermore, stratified analyses were performed limiting the MIS-C patients to those who met the recently revised CDC MIS-C case definition, effective from 1^st^ January 2023: <https://www.cdc.gov/mis/mis-c/hcp_cstecdc/index.html>.

# **Supplementary Results**

## **Healthy controls**

In the RNA-Seq discovery cohort, samples from 134 HC children were included with a median age of 92 months (IQR: 29-154). 47% (*n*=63) of the HC in the discovery cohort were female. Healthy controls were used for normalisation of the data but not for differential expression analysis or feature selection.

In the RT-qPCR cohort there were samples from 24 HC children with a median age of 78 months (IQR: 27-119) and 38% (*n*=9) were female.

## **Principal Component Analysis**

Visualisation of principal components (PCs) from PCA showed that HC and viral patients formed clear distinct clusters when PC1 and PC2 were considered, with MIS-C patients forming a cluster between the KD and bacterial patients (Supplementary Figure 1). Indeed, there is a negative correlation between PC1 scores and whether patients are in the MIS-C (Pearson’s *r*: -0.2), KD (Pearson’s *r*: -0.1) or bacterial (Pearson’s *r*: -0.39) phenotypic groups, and a positive correlation between PC1 scores and viral (Pearson’s *r*: 0.22) or healthy control (Pearson’s *r*: 0.44) groups (Supplementary Figure 2). The maximum correlation between RNA-Seq batch (EUCLIDS or PERFORM RNA-Seq) and any of the PCs was 0.08 (Pearson’s *r*), suggesting that batch effects had successfully been removed. Age was strongly negatively correlated with PC2 (Pearson’s *r*: -0.50) and PC4 (Pearson’s *r*: 0.38), and sex was very strongly negatively correlated with PC6 (Pearson’s *r*: -0.91).

## **RT-qPCR sample exclusions and data analysis**

Samples were excluded if they had missing *GAPDH* measurements in 3 or more of the 4 *GAPDH* assays (*n*=6; 3 x HC, 1 x bacterial, 1 x viral, 1 x KD), or if they were outliers on PCA plots (*n*=2; 1 x MIS-C, 1 x KD). Bacterial patients with COVID-19 coinfection were also removed (*n*=1) along with samples for whom the sample was obtained more than 25 days since the onset of symptoms (*n*=4; 4 x DB).

For each sample, a weighted DRS was calculated as shown in the equation in 2.3.2. The following gene coefficients were used: *HSPBAP1* = -0.69503869; *MX2* = -0.02060998; *TGFB1* = 2.51608739; *VPS37C* = -0.15126113; *TRBV11-2* = -1.54319986.

## **Sub-group analyses exploring the effects of confounding on the 5-gene signature**

Various sub-group analyses were performed to determine whether the performance of the 5-gene signature was influenced by any potentially confounding factors. The following factors were explored in the RT-qPCR validation set:

- Whether patients required inotropic support
- The duration of their symptoms at the time of sampling
- The patients’ age

For each sub-group analysis, the DRS was not re-estimated, but the ROC curves were regenerated for subsets of the patients.

*Inotropic Support*

Out of the MIS-C patients included in the RT-qPCR validation set, 42% (*n*=15) required inotropic support. The proportions of patients in the other phenotypic groups that required inotropic support were much lower: 0% (*n*=0) for KD; 2.4% (*n*=1) for viral; 10% (*n*=5) for bacterial; 2.6% (*n*=1) for COVID-19. We evaluated the possibility that the presence of shock requiring inotropic support caused gene expression changes that then led to the observed performance of the 5-gene signature. To address this potential confounding, the performance of the 5-gene signature was explored in the validation set using individuals who did not require inotropic support (MIS-C *n*=21; KD *n*=17; viral infections *n*=41; bacterial infections *n*=45; COVID-19 *n*=38) using the weighted DRS calculated from the full dataset. ROC curves were compared to the original ROC curve in the RT-qPCR dataset for each comparison using Delong’s test for contrasting ROC curves ^14^. The following AUCs were obtained:

- MIS-C *vs*. all including COVID-19 (KD, viral infections, bacterial infections, COVID-19): 88.0% (95% CI: 80.6%-95.4%) – reduction of 2.8, Delong’s *p*-value: 0.542
- MIS-C *vs*. all excluding COVID-19 (KD, viral infections, bacterial infections): 90.5% (95% CI: 83.5%-97.4%) – reduction of 2.7%, Delong’s *p*-value: 0.515
- MIS-C *vs*. KD: 88.2% (95% CI: 77.3%-99.2%) - reduction of 2.6%, Delong’s *p*-value: 0.716
- MIS-C *vs*. viral infections: 85.1% (95% CI: 74.9%-95.4%) – reduction of 3.9%, Delong’s *p*-value: 0.541
- MIS-C *vs*. bacterial infections: 96.2% (95% CI: 91.8%-100%) – reduction of 1.4%, Delong’s *p*-value: 0.608
- MIS-C *vs*. COVID-19: 81.2% (95% CI: 70.1%-92.3%) – reduction of 2.7%, Delong’s *p*-value: 0.710

Despite the lower performance seen overall and for all sub-group comparisons except for COVID-19, when the ROC curves were compared using Delong’s test for two ROC curves, there were no significant differences in the ROC curves following removal of patients who received inotropes. These findings suggest that although the performance is slightly lower, the distinguishing power of the 5-gene signature is not driven by differences in clinical severity and presence of shock. Of note, the smallest reduction in performance was observed for the MIS-C *vs*. bacterial comparison which is a very important distinction for guiding clinical care.

*Duration of Illness*

Duration of illness (e.g., the number of days that the patient has experienced symptoms upon the day of sampling) is an important consideration to ensure that the patients that are being compared are at a similar stage of disease. Whilst the median day of illness across all patients included in the RT-qPCR validation set was four days (Table 1), there were differences observed between the phenotypic groups with MIS-C patients reporting the longest duration of symptoms (median 5.5 days), followed by KD patients (5 days), viral patients (4 days) and finally bacterial and COVID-19 patients (median 2 days). These differences between groups in duration of illness could drive the observed performance of the 5-gene signature. To explore this possibility, patients were stratified according to whether they had experienced >4 days or <= 4 days of symptoms and sub-group analyses were performed, using the weighted DRS calculated from the full dataset.

The following number of patients had experienced symptoms for <=4 days at the time of sampling: 11 x MIS-C; 6 x KD; 23 x viral infections; 37 x bacterial infections; 29 x COVID-19. The following number of patients had experienced symptoms for >4 days at the time of sampling: 25 x MIS-C; 11 x KD; 19 x viral infections; 13 x bacterial infections; 10 x COVID-19.

ROC curves were generated for each comparison were compared using Delong’s test ^14^. The performance of the 5-gene signature was not significantly different between patients with symptoms >4 or <=4 days for any of the comparisons. All AUCs and Delong’s *p*-values are listed below:

- MIS-C *vs*. all groups including COVID-19 (KD, viral infections, bacterial infections, COVID-19):
  - Patients with illness day >4: 90.4% (95% CI: 82.9%-97.9%)
  - Patients with illness day <=4: 90.1% (95% CI: 83.1%-97.2%)
  - Delong’s *p*-value: 0.959
- MIS-C *vs*. all groups excluding COVID-19 (KD, viral infections, bacterial infections):
  - Patients with illness day >4: 90.1% (95% CI: 82.3%-97.9%)
  - Patients with illness day <=4: 95.3% (95% CI: 90.2%-100%)
  - Delong’s *p*-value: 0.2802
- MIS-C *vs*. KD:
  - Patients with illness day >4: 90.9% (95% CI: 79.2%-100%)
  - Patients with illness day <=4: 89.4 (95% CI: 74.0%-100%)
  - Delong’s *p*-value: 0.879
- MIS-C *vs*. viral infections:
  - Patients with illness day >4: 85.3% (95% CI: 74.0%-96.6%)
  - Patients with illness day <=4: 92.1% (95% CI: 83.2%-100%)
  - Delong’s *p*-value: 0.355
- MIS-C *vs*. bacterial infections:
  - Patients with illness day >4: 96.6% (95% CI: 91.6%-100%)
  - Patients with illness day <=4: 98.3% (95% CI: 95.6%-100%)
  - Delong’s *p*-value: 0.570
- MIS-C *vs*. COVID-19:
  - Patients with illness day >4: 91.6% (95% CI: 82.5%-100%)
  - Patients with illness day <=4: 78.4% (95% CI: 64.0%-92.8%)
  - Delong’s *p*-value: 0.132

The small, insignificant changes in AUC when patients were stratified by illness day suggests that the performance of the 5-gene signature observed in the RT-qPCR validation set is not driven by differences in the duration of illness between MIS-C and the comparator groups.

*Age*

Whilst all patients included in this study were under 18 years old, there were differences between the phenotypic groups with MIS-C children being older (median age of 126 months in the discovery and 103 in the validation). Age was included in the DESeq2 ^7^ models, however, to verify that the 5-gene signature’s performance was not driven by differences in age between phenotypic groups, sub-analyses were performed, stratifying the patients by age.

For MIS-C patients in the validation set, the first quartile of age was 64 months. Therefore, non-MIS-C patients younger than the first quartile were removed, and the performance of the signature was evaluated in patients that were more similar in age to MIS-C patients with the following numbers of patients in each group: MIS-C, *n*=36; bacterial, *n*=20; viral, *n*=13; KD, *n*=4; COVID-19, *n*=15). The AUCs are listed below:

- MIS-C *vs*. all groups excluding COVID-19 (KD, viral, bacterial): 96.7% (95% CI: 93.4%-100%)
- MIS-C *vs*. all groups including COVID-19 (KD, viral, bacterial, COVID-19): 96.3% (95% CI: 93.0%-99.7%)
- MIS-C *vs* KD: 100% (95% CI: 100%-100%)
- MIS-C *vs*. viral: 93.8% (95% CI: 87.1%-100%)
- MIS-C *vs*. bacterial: 97.9% (95% CI: 95.1%-100%)
- MIS-C *vs*. COVID-19: 95.4% (95% CI: 89.9%-100%)

The performance of the signature improved across all comparisons when the samples from younger children were removed, indicating that the 5-gene signature is not purely capturing differences in age between patients in the different phenotypic groups.

## **Performance of the 5-gene signature using MIS-C patients who meet the revised CDC criteria**

The Centers for Disease Control and Prevention (CDC) and The Council of State and Territorial Epidemiologists (CSTE) developed a new MIS-C surveillance case definition to be used from 1^st^ January 2023 (<https://www.cdc.gov/mis/mis-c/hcp_cstecdc/index.html>) which differs slightly from the WHO case definition used in this study. One of the key differences is that the revised CDC definition requires laboratory evidence of SARS-CoV-2 for confirmed MIS-C, unlike the WHO definition which requires evidence of COVID-19 (RT-PCR, antigen test or serology positive), or likely contact with patients with COVID-19. The revised CDC definition also requires that patients meet a specific threshold for C-reactive protein (CRP) of >30 mg/L and are all hospitalised. Both definitions require that patients display at least two manifestation categories, however within these categories the revised CDC definition includes specific thresholds, such as platelet count <150,000 cells/μL for the “hematologic involvement” category.

The performance of the 5-gene signature was explored in the RT-qPCR validation set when limiting MIS-C patients to those who meet the revised CDC criteria for confirmed MIS-C (*n*=30). The weighted DRS calculated from the full dataset was used but ROC curves were re-generated, and Delong’s test was used to contrast the newly generated ROC curves to those generated using the full dataset:

- MIS-C *vs*. all groups excluding COVID-19 (KD, viral, bacterial): 95.6% (95% CI: 92.5%-98.7%)
  - Delong’s *p*-value: 0.403
  - Improvement of 2.3%
- MIS-C *vs*. all groups including COVID-19 (KD, viral, bacterial, COVID-19): 93.2% (95% CI: 89.4%-97.1%)
  - Delong’s *p*-value: 0.443
  - Improvement of 2.5%
- MIS-C *vs*. KD: 93.5% (95% CI: 85.5%-100%)
  - Delong’s *p*-value: 0.661
  - Improvement of 2.7%
- MIS-C *vs*. viral infections: 91.9% (95% CI: 86.0%-97.9%)
  - Delong’s *p*-value: 0.540
  - Improvement of 2.7%
- MIS-C *vs*. bacterial infections: 99.3% (95% CI: 98.3%-100%)
  - Delong’s *p*-value: 0.232
  - Improvement of 1.8%
- MIS-C *vs*. COVID-19: 86.8% (95% CI: 78.5%-95.0%)
  - Delong’s *p*-value: 0.647
  - Improvement of 2.8%

Across all comparisons, limiting MIS-C patients to those who meet the revised CDC criteria for confirmed MIS-C led to higher AUC values for the 5-gene signature, however none of these improvements were significant. This is perhaps unsurprising given the more stringent CDC definition in comparison to the WHO definition, and possibly indicates that some patients categorised as having MIS-C according to the WHO definition may not represent “true” MIS-C. An ongoing limitation of all studies exploring MIS-C is that the lack of a gold standard diagnostic test for MIS-C which means that some MIS-C patients may have been misclassified and may not be “true” MIS-C, thus providing further impetus for an alternative diagnostic approach.

Whilst we categorised patients according to the revised CDC definition to the best of our abilities, the clinical information collected for the patients recruited in our study represent the MIS-C case definition that was used at the time. This means that we do not have the exact granularity required to accurately use the revised CDC case definition. Specifically, the revised CDC case definition requires that gastrointestinal involvement is indicated by either abdominal pain, vomiting or diarrhoea, however the clinical information gathered for the MIS-C patients presented in this study only includes whether or not the patient experienced gastrointestinal symptoms (i.e., yes or no) without information about the exact symptoms experienced. As such, we cannot be certain that all MIS-C patients who experienced gastrointestinal symptoms experienced specifically abdominal pain, vomiting or diarrhoea.

## **Retraining the 5-gene RT-qPCR validation signature weights with COVID-19**

Patients with COVID-19 were not included in the RNA-Seq discovery set. Therefore, when the model weights were retrained in the RT-qPCR validation set, the COVID-19 patients were not included. We explored whether retraining the model weights in the RT-qPCR validation set with COVID-19 patients in addition to KD, viral and bacterial patients affected the performance of the 5-gene signature. Model weights were trained using a generalised logistic regression model (GLM) contrasting MIS-C to KD, viral infections, bacterial infections, and COVID-19. These weights were used to re-estimate the DRS which was used to re-estimate the signature’s performance through ROC curves, with AUCs as follows:

- MIS-C *vs*. all groups including COVID-19 (KD, viral infections, bacterial infections, COVID-19): 91.7% (95% CI: 86.9%-96.6%)
  - improvement of 1%, Delong’s *p*-value: 0.368
- MIS-C *vs*. all groups excluding COVID-19 (KD, viral infections, bacterial infections): 92.3% (95% CI: 87.4%-97.1%)
  - reduction of 0.9%, Delong’s *p*-value: 0.344
- MIS-C *vs*. KD: 90.7% (95% CI: 82.5%-98.9%)
  - reduction of 0.2%, Delong’s *p*-value: 0.922
- MIS-C *vs*. viral infections: 87.2% (95% CI: 79.5%-94.8%)
  - reduction of 1.9%, Delong’s *p*-value: 0.258
- MIS-C *vs*. bacterial infections: 97.1% (95% CI: 94.0%-100%)
  - reduction of 0.5%, Delong’s *p*-value: 0.585
- MIS-C *vs*. COVID-19: 90.3% (83.6%-97.0%)
  - improvement of 6.4%, Delong’s *p*-value: 0.002

For MIS-C *vs*. COVID-19, the Delong’s test returned a significant *p*-value (0.002) when contrasting the ROC curves generated from training the model weights with COVID-19 *vs*. not using COVID-19 to train the model weights. There was an improvement of 6.4% for this specific comparison. The performance of the 5-gene signature in distinguishing MIS-C from all groups combined including COVID-19 (KD, viral infections, bacterial infections, COVID-19) improved by 1% following retraining of model weights with COVID-19 included, however this change was not significantly different. For all other comparisons, the performance of the 5-gene signature did not improve upon retraining of the model weights with COVID-19 in addition to KD, viral and bacterial infections.

## **Sub-analyses contrasting MIS-C to Kawasaki disease**

When MIS-C first emerged in 2020, initial reports likened it to KD ^15,16^. Given the similarities between KD and MIS-C, we performed specific sub-analyses exploring the two diseases in more detail. A total of 4,786 genes were SDE between MIS-C *vs*. KD, with 2,681 and 2,105 genes over- and under-expressed, respectively (Supplementary Figure 3). The top SDE gene was *CD163L1* (CD163 Molecule Like 1; LFC = 4.09; BH-adjusted *p*-value: 2.82e^-28^) which encodes a member of the scavenger receptor cysteine-rich (SRCR) superfamily, a superfamily of receptors that act as pattern recognition receptors in the innate immune system ^17^. *CD163L1* has never been associated with KD, however it has been found to be upregulated in SARS-CoV-2-infected HCN2 cells *vs*. non-infected HCN2 cells ^18^, suggesting that it could be involved in cell entry by SARS-CoV-2.

The performance of the 5-gene signature for MIS-C *vs*. KD in the RT-qPCR validation set was 90.8% (95% CI: 81.6%-100%). The MIS-C patients included in the RT-qPCR validation set were older than the KD patients however, with median ages of 103 months and 26 months, respectively. To evaluate whether this considerable age difference was driving the performance observed, we explored the performance of the 5-gene signature for MIS-C *vs*. KD using age stratified groups. The lower quartile of age for MIS-C patients (discovery: 65 months; validation: 64 months) was selected as a cut off, with KD patients younger than the first quartile of age excluded. For the RT-qPCR validation set, this only left *n*=4 KD patients. When these 4 patients were contrasted against MIS-C (*n*=36) using the 5-gene signature, an AUC of 100% was obtained. Since this high performance could reflect the small sample size for KD, this analysis was repeated the RNA-Seq discovery data, contrasting KD patients older than 65 months (*n*=23) to MIS-C patients (*n*=38). In the discovery set, this comparison led to an AUC of 91.0% (95% CI: 84.1%-97.9%), reflecting a drop of just 2.2% from when all KD patients were used. Overall, this analysis suggests that differences in age between MIS-C and KD patients are not driving the discriminatory performance of the 5-gene signature.

The final sub-analysis performed for MIS-C *vs*. KD was a specific analysis exploring the influence of severity on performance of the 5-gene signature in these groups. The MIS-C patients represented a severely unwell group, with 60.6% (*n*=23) and 44% (*n*=16) of the patients requiring paediatric intensive care unit (PICU) admission during their illness in the discovery and validation sets, respectively. To determine whether differences in severity (i.e., PICU admission) were driving the high performance of the 5-gene signature at distinguishing between MIS-C *vs*. KD in the RT-qPCR set, the MIS-C patients who did not require PICU (*n*=20) were contrasted to the KD patients who did not require PICU (*n*=16), resulting in an AUC of 89.4% (95% CI: 78.4%-100%) with a drop of just 1.4% from when all MIS-C and all KD patients were analysed. This provides evidence that the 5-gene signature can distinguish between KD and MIS-C regardless of severity of illness.

# **Supplementary Figures**

**Supplementary Figure 1:** Principal Component Analysis (PCA) performed on the discovery dataset after normalisation with principal components 1-6 shown. Points represent patients and are coloured by phenotype (A), sex at birth (B), age (C), and RNA-Seq batch (D).


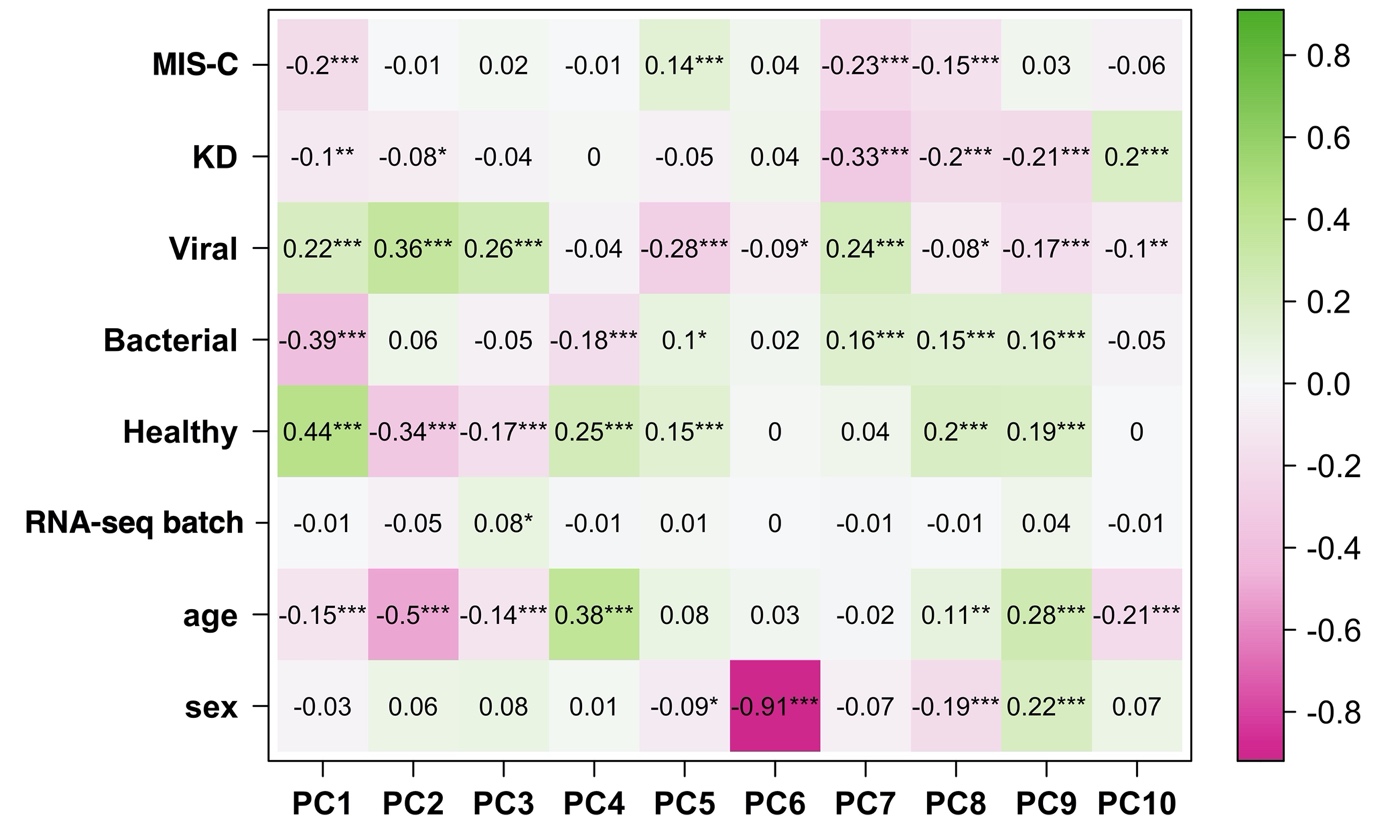


**Supplementary Figure 2:** Eigencorplot generated by PCAtools ^19^. Principal component (PC) scores for each patient are correlated against their metadata values, including whether they are included in any of the phenotypic groups (e.g., 0 = no; 1 = yes). Age is a numeric variable. RNA-Seq batch and sex are binary variables. PCA was performed on the discovery dataset. Correlation (Pearson’s *r*) is shown by the number printed in each cell and the colour of the cell, with the legend shown on the right. Asterix represent the significance of the correlation as follows: *: *p*-value ≤ 0.05; **: *p*-value ≤ 0.01; ***: *p*-value ≤ 0.001.

**Supplementary Figure 3:** Volcano plots showing log2 fold change (LFC) values and adjusted -log10 p-values from differential expression analysis comparing A: MIS-C vs. KD, viral infections, and bacterial infections combined; B: MIS-C vs. KD; C: MIS-C vs. viral infections; D: MIS-C vs. bacterial infection. Points are coloured as followed: red - genes with adjusted p-values <0.05 and absolute LFC >1; yellow – genes with adjusted p-values <0.05 and absolute LFC <1; green – absolute LFC >1; and black = not significant (NS). Genes to the right of 0 are increased in MIS-C patients.

# **Supplementary Tables**

**Supplementary Table 1:** The frequency of causative pathogens identified in the definite bacterial (DB) and definite viral (DV) patient groups in the discovery RNA-Seq cohort and the validation RT-qPCR cohorts.

| **Pathogen** | **Frequency in Discovery** | **Frequency in Validation** |
| --- | --- | --- |
| **Bacteria** | | |
| Acinetobacter spp. | 1 | 0 |
| Bordetella pertussis | 1 | 2 |
| Borrelia burgdorferi | 9 | 3 |
| Campylobacter spp. | 3 | 2 |
| Coagulase negative Staphylococcus | 2 | 0 |
| Enterobacter cloacae | 4 | 1 |
| Enterococcus faecalis | 4 | 1 |
| Enterococcus faecium | 0 | 1 |
| Escherichia coli | 13 | 12 |
| Group A Streptococcus | 17 | 5 |
| Group B Streptococcus | 0 | 2 |
| Haemophilus influenzae | 4 | 2 |
| Klebsiella spp. | 2 | 1 |
| Moraxella catarrhalis | 1 | 0 |
| Neisseria meningitidis | 46 | 5 |
| Prevotella spp. | 1 | 0 |
| Proteus spp. | 2 | 0 |
| Pseudomonas aeruginosa | 4 | 4 |
| Pseudomonas fluorescens | 1 | 0 |
| Salmonella spp. | 4 | 2 |
| Staphylococcus aureus | 30 | 5 |
| Streptococcus pneumoniae | 30 | 7 |
| Streptococcus viridans | 4 | 1 |
| **Virus** | | |
| Adenovirus | 4 | 10 |
| Bocavirus | 2 | 0 |
| Coronavirus (not SARS-CoV-2) | 6 | 0 |
| Dengue virus | 1 | 0 |
| Enterovirus | 6 | 3 |
| Epstein-Barr virus | 2 | 7 |
| Hepatitis A virus | 1 | 2 |
| Herpes simplex virus 1 | 0 | 3 |
| Human metapneumovirus | 1 | 0 |
| Influenza | 7 | 3 |
| Measles | 2 | 4 |
| Norovirus | 0 | 2 |
| Parainfluenza | 3 | 0 |
| Parechovirus | 1 | 0 |
| Parvovirus | 0 | 1 |
| Respiratory syncytial virus | 75 | 6 |
| Rhinovirus | 16 | 1 |
| Rotavirus | 7 | 1 |
| Tick-borne encephalitis | 1 | 0 |
| Varicella zoster virus | 0 | 1 |

**Supplementary Table 2:** Primer sets developed in this study. GAPDH was used as the reference gene.

| **Gene name** | **Exon Ensembl ID** | **forward primer name** | **forward primer sequence** | **reverse primer name** | **reverse primer sequence** |
| --- | --- | --- | --- | --- | --- |
| **HSPBAP1** | ENSE00001900976 | HSPBAP1_forward | CTCCTGTTTCTAGCAAGACTCAA | HSPBAP1_reverse | TTCCCAATTAAGTTCAGCCACT |
| **MX2** | ENSE00003494762 | MX2_forward | GGACGCCTTCACAGAATATGA | MX2_reverse | ATTTCAGTAAAGGAGGAAACCGA |
| **TGFB1** | ENSE00001196164 | TGFB1_forward | ATTTATTGAGCACCTTGGGCA | TGFB1_reverse | TCAGAGTGTTGCTATGGTGAC |
| **TRBV11-2** | ENSE00002493270 | TRBV11-2_forward | ATCTGGCCATGCTACCCTTTA | TRBV11-2_reverse | AAGCTTTGCAGGTTGGATCTT |
| **VPS37C** | ENSE00001119066 | VPS37C_forward | AATTTTCTTCAGCACTGCAGC | VPS37C_reverse | GACTCTTCTTCGATCTTCATGC |
| **GAPDH** | ENSE00000111640 | GAPDH_forward | GACAGTCAGCCGCATCTTC | GAPDH_forward | ACTCCGACCTTCACCTTCC |

# **DIAMONDS, PERFORM and EUCLIDS Consortia**

## **DIAMONDS Consortium**

<https://www.diamonds2020.eu/>

**PARTNER: Imperial College (Coordinating Centre) (UK)**

*Chief investigator/DIAMONDS coordinator:*

Michael Levin^1^

*Principal and co-investigators (alphabetical order)^1^*

Aubrey Cunnington; Jethro Herberg; Myrsini Kaforou; Victoria Wright

*Section of Paediatric Infectious Diseases Research Group (alphabetical order)^1^*

Evangelos Bellos; Claire Broderick; Samuel Channon-Wells; Samantha Cooray; Tisham De (database work package lead); Giselle D’Souza; Leire Estramiana Elorrieta; Diego Estrada-Rivadeneyra; Rachel Galassini (Clinical Trial Manager); Dominic Habgood-Coote; Shea Hamilton (Proteomics); Heather Jackson; James Kavanagh; Mahdi Moradi Marjaneh; Stephanie Menikou; Samuel Nichols; Ruud Nijman; Harsita Patel; Ivana Pennisi; Oliver Powell; Ruth Reid; Priyen Shah; Ortensia Vito; Elizabeth Whittaker; Clare Wilson; Rebecca Womersley

*Recruitment team at Imperial College Healthcare NHS Trust, London (alphabetical order)^2^*

Amina Abdulla; Sarah Darnell; Sobia Mustafa

*Engineering Team*

Pantelis Georgiou^3^ (engineering lead); Jesus-Rodriguez Manzano^4^; Nicolas Moser^3^; Ivana Pennisi^1^

^1^Section of Paediatric Infectious Disease, Imperial College London, Norfolk Place, London W2 1PG, UK

^2^Children’s Clinical Research Unit, St Mary’s Hospital, Praed Street, London W2 1NY, UK

^3^ Imperial College London, Department of Electrical and Electronic Engineering, South Kensington Campus, London, SW7 2AZ, UK

^4^ Imperial College London, Department of Infectious Disease, Section of Adult Infectious Disease, Hammersmith Campus, London, W12 0NN, UK

**UK Non-Consortium Clinical Recruiting Sites**

*Evelina London Children’s Hospital, Guy’s and St Thomas’ NHS Foundation Trust; King’s College London [combined]*

Michael Carter^1,2^ (principal investigator); Shane Tibby^1,2^ (co-investigator)

*Recruitment team (alphabetical order):* Jonathan Cohen^1^; Francesca Davis^1;^ Julia Kenny^1^; Paul Wellman^1^; Marie White^1^

*Laboratory team (alphabetical order):*Matthew Fish^3^; Aislinn Jennings^4^; Manu Shankar-Hari^3,4^

^1^ Evelina London Children’s Hospital, Guy’s and St Thomas’ NHS Foundation Trust, London, UK

^2^Department of Women and Children’s Health, School of Life Course Sciences, King’s College London, UK

^3^Department of Infectious Diseases, School of Immunology and Microbial Sciences, King’s College London, London, UK

^4^Department of Intensive Care Medicine, Guy’s and St Thomas’ NHS Foundation Trust, London, UK

*University Hospitals Sussex*

Katy Fidler^1^  (principal investigator); Dan Agranoff^2^ (co-investigator)

*Recruitment team*; Vivien Richmond^1,3^, Mathhew Seal^2^

^1^ Royal Alexandra Children's Hospital, University Hospitals Sussex, Brighton, UK

^2^ Dept of Infectious Diseases, University Hospitals Sussex, Brighton, UK

^3^ Research Nurse team, University Hospitals Sussex, Brighton, UK

*University Hospital Southampton NHS Foundation Trust*

Saul Faust^1^ (principal investigator); Dan Owen^1^ (co-investigator);

*Recruitment team*; Ruth Ensom^2^; Sarah McKay^2^; Diana Mondo^3^, Mariya Shaji^3^; Rachel Schranz^3^ *(alphabetical order)*

^1^ NIHR Southampton Clinical Research Facility, University Hospital Southampton NHS Foundation Trust and University of Southampton, UK

^2^ NIHR Southampton Clinical Research Facility, University Hospital Southampton NHS Foundation Trust, UK

^3^ Department of R&D, University Hospital Southampton NHS Foundation Trust, UK

*Barts Health NHS Trust*

Prita Rughnani^1, 2, 3^ (principal investigator 2020-2021); Amutha Anpananthar^1, 2, 3^ (principal investigator 2021-to date); Susan Liebeschuetz^2^ (co-investigator), Anna Riddell^1^ (co-investigator)

*Recruitment team;* Nosheen Khalid^1, 3,^Ivone Lancoma Malcolm, Teresa Simagan^3^ *(alphabetical order)*

^1^ Royal London Hospital, Whitechapel Rd, London E1 1FR, UK

^2^ Newham University Hospital, Glen Rd, London E13 8SL, UK

^3^ Whipps Cross University Hospital, Whipps Cross Road, London, E11 1NR, UK

*Great Ormond Street Hospital for Children NHS Foundation Trust*

Mark Peters^1,2^ (principal investigator); Alasdair Bamford^1,2^ (co-investigator)

*Recruitment team;*Lauran O’Neill^1^

^1^ Great Ormond Street Hospital, London, WC1N 3JH, UK

^2^ UCL Great Ormond St Institute of Child Health, WC1N 1EH, UK

*Cambridge University Hospitals NHS Foundation Trust*

Nazima Pathan^1,2^ (principal investigator)

*Recruitment team; Esther Daubney*^1^*, Deborah White*^1^ *(alphabetical order)*

^1^Addenbrooke’s Hospital, Hills Road, Cambridge CB2 0QQ, UK

^2^Department of Paediatrics, University of Cambridge, Cambridge CB2 0QQ, UK

*University College London Hospitals NHS Foundation Trust*

Melissa Heightman^1^ (principal investigator); Sarah Eisen^1^ (co-investigator)

*Recruitment team*; Terry Segal^1^, Lucy Wellings^1^ *(alphabetical order)*

^1^ University College London Hospital, Euston Road, London NW1 2BU, UK

*St George’s University Hospitals NHS Foundation Trust*

Simon B Drysdale^1^ (principal investigator)

*Recruitment team; Nicole Branch*^1^*, Lisa Hamzah*^1^*, Heather Jarman*^1^*(alphabetical order)*

^1^ St George’s Hospital, Blackshaw Road, London SW17 0QT, UK

*Lewisham and Greenwich NHS Trust*

Maggie Nyirenda^1, 2,^(principal investigator)

*Recruitment team* Lisa Capozzi^1^, Emma Gardiner^1^ *(alphabetical order)*

^1^University Hospital Lewisham, London SE13 6LH, UK

^2^ Queen Elizabeth Hospital Greenwich, London SE18 4QH, UK

*Liverpool University Hospitals NHS Foundation Trust*

Robert Moots^1^ (principal investigator); Magda Nasher^2^ (principal investigator)

*Recruitment team*; Anita Hanson^2^; Michelle Linforth^1^

^1^ Aintree University Hospital, Lower Lane, Liverpool L9 7AL, UK

^2^ Royal Liverpool Hospital, Prescot St, Liverpool L7 8XP, UK

*Leeds Teaching Hospitals NHS Trust*

Sean O’Riordan^1^ (principal investigator)

*Recruitment team*; Donna Ellis^1^

^1^Leeds Children’s Hospital, Leeds LS1 3EX, UK

*King’s College Hospital NHS Foundation Trust*

Akash Deep^1^ (principal investigator)

*Recruitment team;* Ivan Caro^1^

^1^ Kings College Hospital, Denmark Hill, London SE5 9RS, UK

*Sheffield Children’s NHS Foundation Trust*

Fiona Shackley ^1^ (principal investigator);

*Recruitment team*; Arianna Bellini,^1^ Stuart Gormley^1^ *(alphabetical order)*

^1^Sheffield Children’s Hospital, Broomhall, Sheffield S10 2TH, UK

*University Hospitals of Leicester NHS Foundation Trust*

Samira Neshat^1^ (principal investigator)

^1^Leicester General Hospital, Leicester LE1 5WW, UK

*Birmingham Women’s and Children’s Hospital NHS Foundation Trust*

Barnaby J Scholefield^1^ (principal investigator)

*Recruitment team; Ceri Robbins*^1^*, Helen Winmill*^1^ *(alphabetical order)*

^1^ Birmingham Children’s Hospital, Steelhouse Lane, Birmingham B4 6NH, UK

**PARTNER: University of Oxford (UK)**

**Children’s Hospital, John Radcliffe Hospital, Oxford**

Principal Investigator

Stéphane C. Paulus^1,2,3^

Co-Principal Investigator

Andrew J. Pollard^1,2,3,4^

Co-investigators

Mark Anthony^1^ (neonates)

Recruitment team

Sarah Hopton^1^, Danielle Miller^1^, Zoe Oliver^1^, Sally Beer^1^, Bryony Ward^1^

^1^John Radcliffe Hospital, Oxford University Hospitals NHS Foundation Trust, Oxford, UK

^2^Department of Paediatrics, University of Oxford, UK

^3^Oxford Vaccine Group, University of Oxford, UK

^4^NIHR Oxford Biomedical Research Centre, Oxford, UK

**University of Oxford, Nepal Site**

Principal Investigator

Shrijana Shrestha^1^

Co-Principal Investigator

Andrew J Pollard^2,3^

**Nepal Research Team**Meeru Gurung^1^
Puja Amatya^1^
Bhishma Pokhrel^1^
Sanjeev Man Bijukchhe^1^

**Oxford Research Team**

Tim Lubinda^2^
Sarah Kelly^2^
Peter O’Reilly^2^

^1^Paediatric Research Unit, Patan Academy of Health Sciences, Kathmandu, Nepal.

^2^Oxford Vaccine Group, Department of Paediatrics, University of Oxford, Oxford, United Kingdom.

^3^NIHR Oxford Biomedical Research Centre, Oxford, United Kingdom.

**PARTNER: SERGAS (Spain)**

Principal Investigators

Federico Martinón-Torres^1^

Antonio Salas^1,2^

GENVIP RESEARCH GROUP (in alphabetical order):

Fernando Álvez González^1^, Xabier Bello^1,2^, Mirian Ben García^1^, Sandra Carnota^1^, Miriam Cebey-López^1^, María José Curras-Tuala^1,2^, Carlos Durán Suárez^1^, Luisa García Vicente^1^, Alberto Gómez-Carballa^1,2^, Jose Gómez Rial^1^, Pilar Leboráns Iglesias^1^, Federico Martinón-Torres^1^, Nazareth Martinón-Torres^1^, José María Martinón Sánchez^1^, Belén Mosquera Pérez^1^, Jacobo Pardo-Seco^1,2^, Lidia Piñeiro Rodríguez^1^, Sara Pischedda^1,2^, Sara Rey Vázquez^1^, Irene Rivero Calle^1^, Carmen Rodríguez-Tenreiro^1^, Lorenzo Redondo-Collazo^1^, Miguel Sadiki Ora^1^, Antonio Salas^1,2^, Sonia Serén Fernández^1^, Cristina Serén Trasorras^1^, Marisol Vilas Iglesias^1^.

^1^ Translational Pediatrics and Infectious Diseases, Pediatrics Department, Hospital Clínico Universitario de Santiago, Santiago de Compostela, Spain, and GENVIP Research Group (www.genvip.org), Instituto de Investigación Sanitaria de Santiago, Universidad de Santiago de Compostela, Galicia, Spain.

^2^ Unidade de Xenética, Departamento de Anatomía Patolóxica e Ciencias Forenses, Instituto de Ciencias Forenses, Facultade de Medicina, Universidade de Santiago de Compostela, and GenPop Research Group, Instituto de Investigaciones Sanitarias (IDIS), Hospital Clínico Universitario de Santiago, Galicia, Spain

^3^ Fundación Pública Galega de Medicina Xenómica, Servizo Galego de Saúde (SERGAS), Instituto de Investigaciones Sanitarias (IDIS), and Grupo de Medicina Xenómica, Centro de Investigación Biomédica en Red de Enfermedades Raras (CIBERER), Universidade de Santiago de Compostela (USC), Santiago de Compostela, Spain

**PARTNER: Liverpool (UK)**

Principal Investigators

Enitan D Carrol^1,2,^

Research Group (in alphabetical order):

Elizabeth Cocklin^1^, Aakash Khanijau^1^, Rebecca Lenihan^1^, Nadia Lewis-Burke^1^

Karen Newall^4^, Sam Romaine^1^, ^1^ Department of Clinical Infection, Microbiology and Immunology, University of Liverpool Institute of Infection, Veterinary and Ecological Sciences , Liverpool, England

^2^ Alder Hey Children’s Hospital, Department of Infectious Diseases, Eaton Road, Liverpool, L12 2AP

^3^ ^4^Alder Hey Children’s Hospital, Clinical Research Business Unit, Eaton Road, Liverpool, L12 2AP

**PARTNER: NATIONAL AND KAPODISTRIAN UNIVERSITY OF ATHENS (Greece)**

Principal Investigator: Maria Tsolia^1^

Co-Investigator: Irini Eleftheriou^1^

PID Unit: Nikos Spyridis^1^, Maria Tambouratzi^1^

Pediatric Rheumatology Unit: Despoina Maritsi^1^

Lab: Antonios Marmarinos^1^, Marietta Xagorari^1^

*Recruitment teams:*

Adult COVID19- Infectious Diseases: Lourida Panagiota, Pefanis Aggelos^2^

Adult COVID19: Akinosoglou Karolina, Gogos Charalambos, Maragos Markos^3^

Adult Inflammatory Diseases-Oncology: Voulgarelis Michalis , Stergiou Ioanna^4^

^1^2^nd^ Department of Pediatrics, National and Kapodistrian University of Athens (NKUA), Children’s Hospital “P, and A. Kyriakou”, Athens, Greece

^2^1^st^ Department of Infectious Diseases, General Hospital “Sotiria”

^3^Pathology Department, University of Patras, General Hospital “Panagia i Voithia”

^4^Pathophysiology Department, Medical Faculty, National and Kapodistrian University of Athens (NKUA), General Hospital “Laiko”

**Newcastle upon Tyne Hospitals NHS Foundation Trust and Newcastle University (UK) combined**

Principal Investigator:

Marieke Emonts ^1,2,3^ (all activities)

Co-investigators

Emma Lim^2,3,6^ (all activities)

John Isaacs^1^ (adult inflammatory)

Recruitment team (alphabetical), datamanagers, and GNCH Research unit:

Kathryn Bell^4^, Stephen Crulley^4^, Daniel Fabian^4^, Evelyn Thomson^4^, Diane Wallia^4^, Caroline Miller^4^  , Ashley Bell^4^

PhD Students/medical staff DIAMONDS

Fabian J.S. van der Velden^1,2^ (all activities), Geoff Shenton^7^ (oncology), Ashley Price^8,9^ (Adult COVID)

Students

Owen Treloar ^1,2^ (quality control, data management and analysis)

Daisy Thomas^1,2^ (recruitment)

Author Affiliations:

^1^ Translational and Clinical Research Institute, Newcastle University, Newcastle upon Tyne UK

^2^Great North Children’s Hospital, Paediatric Immunology, Infectious Diseases & Allergy, Newcastle upon Tyne Hospitals NHS Foundation Trust, Newcastle upon Tyne, United Kingdom.

^3^NIHR Newcastle Biomedical Research Centre based at Newcastle upon Tyne Hospitals NHS Trust and Newcastle University, Westgate Rd, Newcastle upon Tyne NE4 5PL, United Kingdom

^4^Great North Children’s Hospital, Research Unit, Newcastle upon Tyne Hospitals NHS Foundation Trust, Newcastle upon Tyne, United Kingdom.

^6^Population Health Sciences Institute, Newcastle University, Newcastle upon Tyne, UK

^7^Great North Children’s Hospital, Paediatric Oncology, Newcastle upon Tyne Hospitals NHS Foundation Trust, Newcastle upon Tyne, United Kingdom.

^8^Department of Infection & Tropical Medicine, Newcastle upon Tyne Hospitals NHS Foundation Trust, Newcastle upon Tyne, United Kingdom

^9^NIHR Newcastle In Vitro Diagnostics Co-operative (Newcastle MIC), Newcastle upon Tyne, United Kingdom.

**Servicio Madrileño de Salud (SERMAS) - Fundación Biomédica del Hospital Universitario 12 de Octubre (FIB-H12O) (Spain)**

Principal Investigators

Pablo Rojo^1 3^

Cristina Epalza ^1,2^

SERMAS/FIB-H120 team:

Serena Villaverde ^1,^, Sonia Márquez^2^, Manuel Gijón ^2^, Fátima Machín^2^, Laura Cabello^2^, Irene Hernández^2^, Lourdes Gutiérrez^2^, Ángela Manzanares ^1^

Author Affiliations:

^1^ Servicio Madrileño de Salud (SERMAS),Pediatric Infectious Diseases Unit, Department of Pediatrics, Hospital Universitario 12 de Octubre, Madrid, Spain

^2^Fundación Biomédica del Hospital Universitario 12 de Octubre (FIB-H12O), Unidad Pediátrica de Investigación y Ensayos Clínicos (UPIC), Hospital Universitario 12 de Octubre, Instituto de Investigación Sanitaria Hospital 12 de Octubre (i+12), Madrid, Spain.

^3^ Universidad Complutense de Madrid, Faculty of Medicine, Department of Pediatrics, Madrid, Spain.

**Amsterdam University Medical Center (Amsterdam UMC), University of Amsterdam**

Principal Investigator:

T.W. (Taco) Kuijpers MD PhD ^1,2^ (all activities)

Co-investigators

M. (Martijn) van de Kuip MD PhD ^1^ (infectious disease)

A.M. (Marceline) van Furth MD PhD ^1^ (infectious disease)

J.M. (Merlijn) van den Berg MD PhD ^1^ (inflammatory disease)

Hospital Team (all activities):

Giske Biesbroek MD PhD ^1^, Floris Verkuil MD (PhD student) ^1^, Carlijn (C.W.) van der Zee MD (start 1/8/2022, PhD student) ^1^

Recruitment:

Dasja Pajkrt MD PhD ^1^, Michael Boele van Hensbroek MD PhD ^1^, Dieneke Schonenberg MD ^1^, Mariken Gruppen MD ^1^, Sietse Nagelkerke MD PhD ^1,2^, medical students

Laboratory Team:

Machiel H Jansen ^1^, Ines Goetschalckx (PhD student) ^2^

Author Affiliations:

^1^ Amsterdam UMC, Emma Children's Hospital, Dept of Pediatric Immunology, Rheumatology and Infectious Disease, University of Amsterdam, The Netherlands

^2^ Sanquin, Dept of Molecular Hematology, University Medical Center, Amsterdam, The Netherlands

**Bambino Gesù Children’s Hospital (Rome-Italy)**

Principal Investigator

Lorenza Romani 1
Maia De Luca 1

Recruitment Team

Sara Chiurchiù 1
Martina Di Giuseppe 1

Affiliation

1 Infectious Disease Unit, Academic Department of Pediatrics, Bambino Gesù Children's Hospital, IRCCS, Rome 00165, Italy

**ERASMUS MC-Sophia Children’s Hospital**

*Principal Investigator*

Clementien L. Vermont²

*Research group*

Henriëtte A. Moll¹, Dorine M. Borensztajn¹, Nienke N. Hagedoorn, Chantal Tan ¹, Joany Zachariasse ¹, Medical students ¹

Additional investigator

W Dik ^3^

¹ Erasmus MC-Sophia Children’s Hospital, Department of General Paediatrics, Rotterdam, the Netherlands

² Erasmus MC-Sophia Children’s Hospital, Department of Paediatric Infectious Diseases & Immunology, Rotterdam, the Netherlands

^3^ Erasmus MC, Department of immunology, Rotterdam, the Netherlands

**TAIWAN**

Ching-Fen (Kitty), Shen

Division of Infectious Disease, Department of Pediatrics,  National Cheng Kung University

Tainan, Taiwan

**Riga Stradins University (Riga, Latvia)**

Principal Investigator:

Dace Zavadska ^1,2^ (all activities)

Co-investigators

Sniedze Laivacuma ^1,3^ (adult cohorts)

Recruitment team:

Aleksandra Rudzate ^1,2^, Diana Stoldere ^1,2^, Arta Barzdina ^1,2^, Elza Barzdina ^1,2^, Sniedze Laivacuma^1,3^, Monta Madelane ^1,3^

Laboratory

Dagne Gravele ^2^, Dace Svile^2^

Author Affiliations:

^1^ Riga Stradins University, Riga, Latvia

^2^ Children clinical university hospital, Riga, Latvia

^3^ Riga East clinical university hospital, Riga, Latvia

**Assistance Publique - Hôpitaux de Paris**

Principal Investigator:

Romain Basmaci ^1,2^

Co-investigator:

Noémie Lachaume ^1^

Recruitment team:

Pauline Bories ^1^, Raja Ben Tkhayat ^1^, Laura Chériaux ^1^, Juraté Davoust ^1^, Kim-Thanh Ong ^1^, Marie Cotillon ^1^, Thibault de Groc ^1^, Sébastien Le ^1^, Nathalie Vergnault ^1^, Hélène Sée ^1^, Laure Cohen ^1^, Alice de Tugny ^1^, Nevena Danekova ^1^

Author Affiliations:

^1^ Service de Pédiatrie-Urgences, AP-HP, Hôpital Louis-Mourier, F-92700 Colombes, France

² Université Paris Cité, Inserm, IAME, F-75018 Paris, France

**BioMérieux**

Principal Investigator:

Marine Mommert-Tripon

Co-investigator:

Karen Brengel-Pesce

Author Affiliations:

bioMérieux - Open Innovation & Partnerships Department, Lyon, France

**University Medical Centre Ljubljana, Slovenia**

Principal Investigator: Marko Pokorn ^1,2,3^

Co-Investigator: Mojca Kolnik^2^

Research Group (in alphabetical order):

Tadej Avčin^2,3^, Tanja Avramoska^2^, Natalija Bahovec^1^, Petra Bogovič^1^, Lidija Kitanovski^2,3^, Mirijam Nahtigal^1^, Lea Papst^1^, Tina Plankar Srovin^1^, Franc Strle^1,2^, Anja Srpčič^2^, Katarina Vincek^1^.

Affiliations:

1. Department of Infectious diseases, University Medical Centre Ljubljana, Slovenia
2. University Children's Hospital, University Medical Centre Ljubljana, Slovenia
3. Faculty of Medicine, University of Ljubljana, Slovenia
4. Centre for Clinical research, University Medical Centre Ljubljana

**University Medical Center Utrecht, Utrecht, The Netherlands**

*Principal Investigator*

Michiel van der Flier^1,5 (^Pediatric Infectious Diseases and Immunology)

*Co-investigators*

Wim J.E. Tissing^5^ (Pediatric Oncology)

Roelie M. Wösten-van Asperen^2^ (Pediatric Intensive Care Unit)

Sebastiaan J Vastert^3^ (Pediatric Rheumatology)

Daniel C Vijlbrief^4^ (Pediatric Neonatal Intensive Care)

Louis J. Bont^1,5 (^Pediatric Infectious Diseases and Immunology)

Tom F.W. Wolfs ^1,5 (^Pediatric Infectious Diseases and Immunology)

*PhD student*

Coco R. Beudeker^1,5 (^Pediatric Infectious Diseases and Immunology)

*Affiliations:*

1.Pediatric Infectious Diseases and Immunology, 2. Pediatric Intensive Care Unit 3. Pediatric Rheumatology 4. Pediatric Neonatal Intensive Care, Wilhelmina Children’s Hospital, University Medical Center Utrecht, Utrecht, The Netherlands

5. Princess Maxima Center for Pediatric Oncology, Utrecht, The Netherlands

**PARTNER**: **University of Bern, Inselspital, Bern University Hospital, University of Bern (Switzerland)**

Principal Investigators (alphabetical)

Philipp Agyeman^1^

Luregn Schlapbach^2,3^

Co-Investigator

Christoph Aebi^1^

Recruitment team

Mariama Usman^1^, Stefanie Schlüchter^1^, Verena Wyss^1^, Nina Schöbi^1^, Elisa Zimmermann^2^ PhD, Marion Meier^2^, Kathrin Weber^2^

^1^ Department of Pediatrics, Inselspital, Bern University Hospital, University of Bern, Switzerland

^2^ Department of Intensive Care and Neonatology, and Children`s Research Center, University Children`s Hospital Zurich, Zurich, Switzerland

^3^ Child Health Research Centre, The University of Queensland, Brisbane, Australia

**Swiss Pediatric Sepsis Study group**

Philipp Agyeman, MD ^1^, Luregn J Schlapbach, MD, FCICM ^2,3^, Eric Giannoni, MD ^4,5^, Martin Stocker, MD ^6^, Klara M Posfay-Barbe, MD ^7^, Ulrich Heininger, MD ^8^, Sara Bernhard-Stirnemann, MD ^9^, Anita Niederer-Loher, MD ^10^, Christian Kahlert, MD ^10^, Giancarlo Natalucci, MD ^11^, Christa Relly, MD ^12^, Thomas Riedel, MD ^13^, Christoph Aebi, MD ^1^, Christoph Berger, MD ^12^

**Affiliations:**

^1^ Department of Pediatrics, Inselspital, Bern University Hospital, University of Bern, Switzerland

^2^ Department of Intensive Care and Neonatology, and Children`s Research Center, University Children`s Hospital Zurich, Zurich, Switzerland

^3^ Child Health Research Centre, The University of Queensland, Brisbane, Australia

^4^ Clinic of Neonatology, Department Mother-Woman-Child, Lausanne University Hospital and University of Lausanne, Switzerland

^5^ Infectious Diseases Service, Department of Medicine, Lausanne University Hospital and University of Lausanne, Switzerland

^6^ Department of Pediatrics, Children’s Hospital Lucerne, Lucerne, Switzerland

^7^ Pediatric Infectious Diseases Unit, Children’s Hospital of Geneva, University Hospitals of Geneva, Geneva, Switzerland

^8^ Infectious Diseases and Vaccinology, University of Basel Children’s Hospital, Basel, Switzerland

^9^ Children’s Hospital Aarau, Aarau, Switzerland

^10^ Division of Infectious Diseases and Hospital Epidemiology, Children’s Hospital of Eastern Switzerland St. Gallen, St. Gallen, Switzerland

^11^ Department of Neonatology, University Hospital Zurich, Zurich, Switzerland

^12^ Division of Infectious Diseases and Hospital Epidemiology, and Children’s Research Center, University Children’s Hospital Zurich, Switzerland

^13^ Children’s Hospital Chur, Chur, Switzerland

**Micropathology Ltd (UK)**

Micropathology Ltd, The Venture Center, University of Warwick Science Park, Sir William Lyons Road, Coventry, CV4 7EZ

Principle Investigator; Prof Colin Fink

Co Investigators: Marie Voice, Leo Calvo-Bado, Michael Steele, Jennifer Holden

Research group: Benjamin Evans, Jake Stevens, Peter Matthews, Kyle Billing

**Medical University of Graz, Austria (MUG)**

Principal Investigator:

Werner Zenz^1^ (all activities)

Co-investigators (in alphabetical order):

Alexander Binder^1^ (grant application)

Benno Kohlmaier^1^ (study design, recruitment)

Daniela S. Kohlfürst^1^ (study design)

Nina A. Schweintzger^1^ (all activities)

Christoph Zurl^1^ (study design, recruitment)

Recruitment team, data managers, laboratory work (in alphabetical order):

Susanne Hösele^1^, Manuel Leitner^1^, Lena Pölz^1^, Alexandra Rusu^1^, Glorija Rajic^1^, Bianca Stoiser^1^, Martina Strempfl^1,^ Manfred G. Sagmeister^1^

Clinical recruitment partners (in alphabetical order):

Sebastian Bauchinger^1^, Martin Benesch^3^, Astrid Ceolotto^1^, Ernst Eber^2^, Siegfried Gallistl^1^, Harald Haidl^1^, Almuthe Hauer^1^, Christa Hude^1^, Andreas Kapper^7^, Markus Keldorfer^5^, Sabine Löffler^5^, Tobias Niedrist^6^, Heidemarie Pilch^5^, Andreas Pfleger^2^, Klaus Pfurtscheller^4^, Siegfried Rödl^4^, Andrea Skrabl-Baumgartner^1^, Volker Strenger^3^, Elmar Wallner^7^

Author Affiliations:

^1^ Department of Pediatrics and Adolescent Medicine, Division of General Pediatrics, Medical University of Graz, Graz, Austria

^2^Department of Pediatric Pulmonology, Medical University of Graz, Graz, Austria

^3^Department of Pediatric Hematooncology, Medical University of Graz, Graz, Austria

^4^Paediatric Intensive Care Unit, Medical University of Graz, Graz, Austria

^5^University Clinic of Pediatrics and Adolescent Medicine Graz, Medical University Graz, Graz, Austria

^6^Clinical Institute of Medical and Chemical Laboratory Diagnostics, Medical University Graz, Graz, Austria

^7^Department of Internal Medicine, State Hospital Graz II, Location West, Graz, Austria

**SkylineDX**

Principle investigator: Dennie Tempel ^1^

Co-investigators: Danielle van Keulen^1^, Annelieke M Strijbosch ^1^,

Author affiliations:

^1^ SkylineDx, Rotterdam, The Netherlands

**Project partner BBMRI-ERIC**

Maike K. Tauchert

Author affiliation:

Biobanking and BioMolecular Resources Research Infrastructure - European Research Infrastructure Consortium (BBMRI-ERIC), Neue Stiftingtalstrasse 2/B/6, 8010, Graz, Austria

**LMU Munich Partner (Germany)**

Principal Investigator:

Ulrich von Both^1,2^ MD, FRCPCH (all activities)

Research group:

Laura Kolberg¹ MSc (all activities)

Patricia Schmied¹ (Study physician), Irene Alba-Alejandre^3^ MD (Study physician)

Clinical recruitment partners (in alphabetical order):

Katharina Danhauser, MD^6^, Nikolaus Haas, MD^11^, Florian Hoffmann, MD^10^, Matthias Griese, MD^7^, Tobias Feuchtinger, MD^5^, Sabrina Juranek, MD^4^, Matthias Kappler, MD^7^, Eberhard Lurz, MD^8^, Esther Maier, MD^4^, Karl Reiter, MD^10^, Carola Schoen, MD^10^, Sebastian Schroepf, MD^9^

Author Affiliations:

¹ Division of Pediatric Infectious Diseases, Department of Pediatrics, Dr. von Hauner Children’s Hospital, University Hospital, LMU Munich, Munich, Germany

^2^ German Center for Infection Research (DZIF), Partner Site Munich, Munich, Germany

^3^ Department of Gynecology and Obstetrics, University Hospital, LMU Munich, Munich, Germany

^4^ Division of General Pediatrics, Department of Pediatrics, Dr. von Hauner Children’s Hospital, University Hospital, LMU Munich, Munich, Germany

^5^ Division of Pediatric Haematology & Oncology, Department of Pediatrics, Dr. von Hauner Children’s Hospital, University Hospital, LMU Munich, Munich, Germany

^6^ Division of Pediatric Rheumatology, Department of Pediatrics, Dr. von Hauner Children’s Hospital, University Hospital, LMU Munich, Munich, Germany

^7^ Division of Pediatric Pulmonology, Department of Pediatrics, Dr. von Hauner Children’s Hospital, University Hospital, LMU Munich, Munich, Germany

^8^ Division of Pediatric Gastroenterology, Department of Pediatrics, Dr. von Hauner Children’s Hospital, University Hospital, LMU Munich, Munich, Germany

^9^ Neonatal Intensive Care Unit, Department of Pediatrics, Dr. von Hauner Children’s Hospital, University Hospital, LMU Munich, Munich, Germany

^10^ Paediatric Intensive Care Unit, Department of Pediatrics, Dr. von Hauner Children’s Hospital, University Hospital, LMU Munich, Munich, Germany

^11^ Department of Pediatric Cardiology and Pediatric Intensive Care, University Hospital, LMU Munich, Germany

**London School of Hygiene and Tropical Medicine (LSHTM)**

Principal Investigator: Shunmay Yeung ^1,2,3^

Research group:

Manuel Dewez^1^ David Bath^3^, Elizabeth Fitchett^1^, Fiona Cresswell^1^

1. Clinical Research Department, Faculty of Infectious and Tropical Disease, London School of Hygiene and Tropical Medicine, London
2. Department of Paediatrics, St. Mary’s Imperial College Hospital, London
3. Department of Global Health and Development, Faculty of Public Health and Policy, London School of Hygiene and Tropical Medicine, London

## **PERFORM Consortium**

<https://www>.perform2020.org/

**PARTNER: IMPERIAL COLLEGE (UK)**

Chief investigator/PERFORM coordinator:

Michael Levin

Principal and co-investigators; work package leads (alphabetical order)

Aubrey Cunnington (grant application)

Tisham De (work package lead)

Jethro Herberg (Principle Investigator, Deputy Coordinator, grant application)

Myrsini Kaforou (grant application, work package lead)

Victoria Wright (grant application, Scientific Coordinator)

Research Group (alphabetical order)

Lucas Baumard; Evangelos Bellos; Giselle D’Souza; Rachel Galassini; Dominic Habgood-Coote; Shea Hamilton; Clive Hoggart; Sara Hourmat; Heather Jackson; Ian Maconochie; Stephanie Menikou; Naomi Lin; Samuel Nichols; Ruud Nijman; Ivonne Pena Paz; Oliver Powell, Priyen Shah; Ching-Fen Shen; Clare Wilson

Clinical recruitment at Imperial College Healthcare NHS Trust (alphabetical order)

Amina Abdulla; Ladan Ali; Sarah Darnell; Rikke Jorgensen; Sobia Mustafa; Salina Persand

Imperial College Faculty of Engineering

Molly Stevens (co-investigator), Eunjung Kim (research group); Benjamin Pierce (research group)

Clinical recruitment at Brighton and Sussex University Hospitals

Katy Fidler (Principle Investigator)

Julia Dudley (Clinical Research Registrar)

Research nurses: Vivien Richmond, Emma Tavliavini

Clinical recruitment at National Cheng Kung University Hospital

Ching-Fen Shen (Principal Investigator); Ching-Chuan Liu (Co-investigator); Shih-Min Wang (Co-investigator), funded by the Center of Clinical Medicine Research, National Cheng Kung University

**PARTNER: SERGAS (Spain)**

Principal Investigators

Federico Martinón-Torres^1^

Antonio Salas^1,2^

Research Group (alphabetical order)

Fernando Álvez González^1^, Cristina Balo Farto^1^, Ruth Barral-Arca^1,2^, María Barreiro Castro^1^, Xabier Bello^1,2^, Mirian Ben García^1^, Sandra Carnota^1^, Miriam Cebey-López^1^, María José Curras-Tuala^1,2^, Carlos Durán Suárez^1^, Luisa García Vicente^1^, Alberto Gómez-Carballa^1,2^, Jose Gómez Rial^1^, Pilar Leboráns Iglesias^1^, Federico Martinón-Torres^1^, Nazareth Martinón-Torres^1^, José María Martinón Sánchez^1^, Belén Mosquera Pérez^1^, Jacobo Pardo-Seco^1,2^, Lidia Piñeiro Rodríguez^1^, Sara Pischedda^1,2^, Sara Rey Vázquez^1^, Irene Rivero Calle^1^, Carmen Rodríguez-Tenreiro^1^, Lorenzo Redondo-Collazo^1^, Miguel Sadiki Ora^1^, Antonio Salas^1,2^, Sonia Serén Fernández^1^, Cristina Serén Trasorras^1^, Marisol Vilas Iglesias^1^.

^1^ Translational Pediatrics and Infectious Diseases, Pediatrics Department, Hospital Clínico Universitario de Santiago, Santiago de Compostela, Spain, and GENVIP Research Group ([www.genvip](http://www.genvip).org), Instituto de Investigación Sanitaria de Santiago, Universidad de Santiago de Compostela, Galicia, Spain.

^2^ Unidade de Xenética, Departamento de Anatomía Patolóxica e Ciencias Forenses, Instituto de Ciencias Forenses, Facultade de Medicina, Universidade de Santiago de Compostela, and GenPop Research Group, Instituto de Investigaciones Sanitarias (IDIS), Hospital Clínico Universitario de Santiago, Galicia, Spain

^3^ Fundación Pública Galega de Medicina Xenómica, Servizo Galego de Saúde (SERGAS), Instituto de Investigaciones Sanitarias (IDIS), and Grupo de Medicina Xenómica, Centro de Investigación Biomédica en Red de Enfermedades Raras (CIBERER), Universidade de Santiago de Compostela (USC), Santiago de Compostela, Spain

**PARTNER: RSU (Latvia)**

Principal Investigator

Dace Zavadska^1,2^

Other RSU group authors (in alphabetical order):

Anda Balode^1,2^, Arta Bārzdiņa^1,2^, Dārta Deksne^1,2^, Dace Gardovska^1,2^, Dagne Grāvele^2^, Ilze Grope^1,2^, Anija Meiere^1,2^, Ieva Nokalna^1,2^, Jana Pavāre^1,2^, Zanda Pučuka^1,2^, Katrīna Selecka^1,2^, Aleksandra Sidorova^1,2^, Dace Svile^2^, Urzula Nora Urbāne^1,2^.

^1^ Riga Stradins university, Riga, Latvia.

^2^ Children clinical university hospital, Riga, Latvia.

**PARTNER: Medical Research Council Unit The Gambia (MRCG) at LSHTM**

Principal Investigator

Effua Usuf

Additional Investigators

Kalifa Bojang

Syed M. A. Zaman

Fatou Secka

Suzanne Anderson

Anna RocaIsatou Sarr

Momodou Saidykhan

Saffiatou Darboe

Samba Ceesay

Umberto D’alessandro

Medical Research Council Unit The Gambia at LSHTM

P O Box 273,

Fajara, The Gambia

**PARTNER: ERASMUS MC-Sophia Children’s Hospital (Netherlands**

Principal Investigator

Henriëtte A. Moll¹

Research Group (alphabetical order)

Dorine M. Borensztajn¹, Nienke N. Hagedoorn, Chantal Tan ¹, ¹, Clementien L. Vermont², Joany Zachariasse ¹

Additional investigator

W Dik ^3^

¹ Erasmus MC-Sophia Children’s Hospital, Department of General Paediatrics, Rotterdam, the Netherlands

² Erasmus MC-Sophia Children’s Hospital, Department of Paediatric Infectious Diseases & Immunology, Rotterdam, the Netherlands

^3^ Erasmus MC, Department of immunology, Rotterdam, the Netherlands

**PARTNER: Swiss Pediatric Sepsis Study (Switzerland)**

Principal Investigators*:*

Philipp Agyeman, MD ^1^ (ORCID 0000-0002-8339-5444), Luregn J Schlapbach, MD, FCICM ^2,3^ (ORCID 0000-0003-2281-2598)

Clinical recruitment at University Children’s Hospital Bern for PERFORM:

Christoph Aebi ^1^, Verena Wyss ^1^, Mariama Usman ^1^

Principal and co-investigators for the Swiss Pediatric Sepsis Study:

Philipp Agyeman, MD ^1^, Luregn J Schlapbach, MD, FCICM ^2,3^, Eric Giannoni, MD ^4,5^, Martin Stocker, MD ^6^, Klara M Posfay-Barbe, MD ^7^, Ulrich Heininger, MD ^8^, Sara Bernhard-Stirnemann, MD ^9^, Anita Niederer-Loher, MD ^10^, Christian Kahlert, MD ^10^, Giancarlo Natalucci, MD ^11^, Christa Relly, MD ^12^, Thomas Riedel, MD ^13^, Christoph Aebi, MD ^1^, Christoph Berger, MD ^12^ for the Swiss Pediatric Sepsis Study

^1^ Department of Pediatrics, Inselspital, Bern University Hospital, University of Bern, Switzerland

^2^ Neonatal and Pediatric Intensive Care Unit, Children’s Research Center, University Children’s Hospital Zurich, University of Zurich, Zurich, Switzerland

^3^Child Health Research Centre, University of Queensland, and Queensland Children`s Hospital, Brisbane, Australia

^4^ Clinic of Neonatology, Department Mother-Woman-Child, Lausanne University Hospital and University of Lausanne, Switzerland

^5^ Infectious Diseases Service, Department of Medicine, Lausanne University Hospital and University of Lausanne, Switzerland

^6^ Department of Pediatrics, Children’s Hospital Lucerne, Lucerne, Switzerland

^7^ Pediatric Infectious Diseases Unit, Children’s Hospital of Geneva, University Hospitals of Geneva, Geneva, Switzerland

^8^ Infectious Diseases and Vaccinology, University of Basel Children’s Hospital, Basel, Switzerland

^9^ Children’s Hospital Aarau, Aarau, Switzerland

^10^ Division of Infectious Diseases and Hospital Epidemiology, Children’s Hospital of Eastern Switzerland St. Gallen, St. Gallen, Switzerland

^11^ Department of Neonatology, University Hospital Zurich, Zurich, Switzerland

^12^ Division of Infectious Diseases and Hospital Epidemiology, and Children’s Research Center, University Children’s Hospital Zurich, Switzerland

^13^ Children’s Hospital Chur, Chur, Switzerland

**PARTNER: Liverpool (UK)**

Principal Investigators

Enitan D Carrol^1,2,3^

Stéphane Paulus ^1,^

Research Group (alphabetical order)

Elizabeth Cocklin^1^, Rebecca Jennings^4^, Joanne Johnston^4^, Simon Leigh^1^, Karen Newall^4^, Sam Romaine^1^

^1^ Department of Clinical Infection, Microbiology and Immunology, University of Liverpool Institute of Infection and Global Health , Liverpool, England

^2^ Alder Hey Children’s Hospital, Department of Infectious Diseases, Eaton Road, Liverpool, L12 2AP

^3^ Liverpool Health Partners, 1^st^ Floor, Liverpool Science Park, 131 Mount Pleasant, Liverpool, L3 5TF

^4^Alder Hey Children’s Hospital, Clinical Research Business Unit, Eaton Road, Liverpool, L12 2AP

**PARTNER: NKUA (Greece)**

Principal investigator

Professor Maria Tsolia (all activities)

Investigator/Research fellow

Irini Eleftheriou (all activities)

Additional investigators

Recruitment: Maria Tambouratzi

Lab: Antonis Marmarinos (Quality Manager)

Lab: Marietta Xagorari

Kelly Syggelou

2^nd^ Department of Pediatrics, National and Kapodistrian University of Athens,

“P. and A. Kyriakou” Children’s Hospital

Thivon and Levadias

Goudi, Athens

**PARTNER : Micropathology Ltd (UK)**

Principal Investigator

Professor Colin Fink^1^, Clinical Microbiologist

Additional investigators

Dr Marie Voice^1^, Post doc scientist

Dr. Leo Calvo-Bado^1^, Post doc scientist

^1^ Micropathology Ltd, The Venture Center, University of Warwick Science Park, Sir William Lyons Road, Coventry, CV4 7EZ.

**PARTNER : Medical University of Graz (MUG, Austria)**

Principal Investigator

Werner Zenz^1^ (all activities)

Co-investigators (alphabetical order)

Benno Kohlmaier^1^ (all activities)

Nina A. Schweintzger^1^ (all activities)

Manfred G. Sagmeister^1^ (study design, consortium wide sample management)

Research team

Daniela S. Kohlfürst^1^ (study design)

Christoph Zurl^1^ (BIVA PIC)

Alexander Binder^1^ (grant application)

Recruitment team, data managers, (alphabetical order)

Susanne Hösele^1^, Manuel Leitner^1^, Lena Pölz^1^, Glorija Rajic^1^,

Clinical recruitment partners (alphabetical order)

Sebastian Bauchinger^1^, Hinrich Baumgart^4^, Martin Benesch^3^, Astrid Ceolotto^1^, Ernst Eber^2^, Siegfried Gallistl^1^, Gunther Gores^5^, Harald Haidl^1^, Almuthe Hauer^1^, Christa Hude^1^, Markus Keldorfer^5^, Larissa Krenn^4^, Heidemarie Pilch^5^, Andreas Pfleger^2^, Klaus Pfurtscheller^4^, Gudrun Nordberg^5^, Tobias Niedrist^8^, Siegfried Rödl^4^, Andrea Skrabl-Baumgartner^1^, Matthias Sperl^7^, Laura Stampfer^5^, Volker Strenger^3^, Holger Till^6^, Andreas Trobisch^5^, Sabine Löffler^5^

^1^ Department of Pediatrics and Adolescent Medicine, Division of General Pediatrics, Medical University of Graz, Graz, Austria

^2^Department of Pediatric Pulmonology, Medical University of Graz, Graz, Austria

^3^Department of Pediatric Hematooncoloy, Medical University of Graz, Graz, Austria

^4^Paediatric Intensive Care Unit, Medical University of Graz, Graz, Austria

^5^University Clinic of Paediatrics and Adolescent Medicine Graz, Medical University Graz, Graz,Austria

^6^Department of Paediatric and Adolescence Surgery, Medical University Graz, Graz, Austria

^7^Department of Pediatric Orthopedics, Medical University Graz, Graz, Austria

^8^Clinical Institute of Medical and Chemical Laboratory Diagnostics, Medical University Graz, Graz, Austria

**PARTNER: London School of Hygiene and Tropical Medicine (UK)**

WP 1 WP2, WP5

Principal Investigator:

Dr Shunmay Yeung^1,2 3^ PhD, MBBS, FRCPCH, MRCP, DTM&H

Research Group

Dr Juan Emmanuel Dewez^1^ MD, DTM&H, MSc

Prof Martin Hibberd ^1^ BSc, PhD

Mr David Bath^2^ MSc, MappFin, BA(Hons)

Dr Alec Miners^2^ BA(Hons), MSc, PhD

Dr Ruud Nijman^3^ PhD MSc MD MRCPCH

Dr Catherine Wedderburn^1^ BA, MBChB, DTM&H, MSc, MRCPCH

Ms Anne Meierford^1^ MSc, BmedSc, BMBS

Dr Baptiste Leurent^4^, PhD, MSc

1. Faculty of Infectious and Tropical Disease, London School of Hygiene and Tropical Medicine, London, UK
2. Faculty of Public Health and Policy, London School of Hygiene and Tropical Medicine, London, UK
3. Department of Paediatrics, St. Mary’s Hospital Imperial College Hospital, London, UK
4. Faculty of Epidemiology and Population Health, London School of Hygiene and Tropical Medicine, London, UK

**PARTNER: Radboud University Medical Center (RUMC, Netherlands)**

Principal Investigators

Ronald de Groot ^1^, Michiel van der Flier ^1,2,3^, Marien I. de Jonge^1^

Co-investigators Radboud University Medical Center (alphabetical order)

Koen van Aerde^1,2^, Wynand Alkema^1^, Bryan van den Broek^1^, Jolein Gloerich^1^, Alain J. van Gool^1^, Stefanie Henriet^1,2^, Martijn Huijnen^1^, Ria Philipsen^1^, Esther Willems^1^

Investigators PeDBIG PERFORM DUTCH CLINICAL NETWORK (alphabetical order)

G.P.J.M. Gerrits^8^, M. van Leur^8,^ J. Heidema ^4^,L. de Haan^1,2^ C.J. Miedema ^5^, C. Neeleman ^1^ C.C. Obihara ^6^, G.A. Tramper-Stranders7^6^

1. Radboud University Medical Center, Nijmegen, The Netherlands
2. Amalia Children’s Hospital, Nijmegen, The Netherlands
3. Wilhelmina Children’s Hospital, University Medical Center Utrecht, Utrecht, The Netherlands
4. St. Antonius Hospital, Nieuwegein, The Netherlands
5. Catharina Hospital, Eindhoven, The Netherlands
6. ETZ Elisabeth, Tilburg, The Netherlands
7. Franciscus Gasthuis, Rotterdam, The Netherlands
8. Canisius Wilhelmina Hospital, Nijmegen, The Netherlands

**PARTNER: Oxford (UK)**

Principal Investigators

Andrew J. Pollard^1,2^, Rama Kandasamy^1,2^, Stéphane Paulus ^1,2^

Additional Investigators

Michael J. Carter^1,2^, Daniel O’Connor^1,2^, Sagida Bibi^1,2^, Dominic F. Kelly^1,2^, Meeru Gurung^3^, Stephen Thorson^3^, Imran Ansari^3^, David R. Murdoch^4^, Shrijana Shrestha^3^.

^1^Oxford Vaccine Group, Department of Paediatrics, University of Oxford, Oxford, United Kingdom.

^2^NIHR Oxford Biomedical Research Centre, Oxford, United Kingdom.

^3^Paediatric Research Unit, Patan Academy of Health Sciences, Kathmandu, Nepal.

^4^Department of Pathology, University of Otago, Christchurch, New Zealand.

**PARTNER: Newcastle University, Newcastle upon Tyne, (UK)**

Principal Investigator

Marieke Emonts ^1,2,3^ (all activities)

Co-investigators

Emma Lim^2,3,7^ (all activities)

Lucille Valentine^4^

Recruitment team (alphabetical), data-managers, and GNCH Research unit

Karen Allen^5^, Kathryn Bell^5^, Adora Chan^5^, Stephen Crulley^5^, Kirsty Devine^5^, Daniel Fabian^5^, Sharon King^5^, Paul McAlinden^5^, Sam McDonald^5^, Anne McDonnell2,^5^, Ailsa Pickering^2,5^, Evelyn Thomson^5^, Amanda Wood^5^, Diane Wallia^5^, Phil Woodsford^5^,

Sample processing: Frances Baxter^5^, Ashley Bell^5^, Mathew Rhodes^5^

PICU recruitment

Rachel Agbeko^8^

Christine Mackerness^8^

Students MOFICHE

Bryan Baas^2^, Lieke Kloosterhuis^2^, Wilma Oosthoek^2^

Students/medical staff PERFORM

Tasnim Arif^6^, Joshua Bennet^2^, Kalvin Collings^2^, Ilona van der Giessen^2^, Alex Martin^2^, Aqeela Rashid^6^, Emily Rowlands^2^, Gabriella de Vries^2^, Fabian van der Velden^2^

Engagement work/ethics/cost effectiveness

Lucille Valentine ^4^, Mike Martin^9^, Ravi Mistry^2^, Lucille Valentine^4^

^1^ Translational and Clinical Research Institute, Newcastle University, Newcastle upon Tyne UK

^2^Great North Children’s Hospital, Paediatric Immunology, Infectious Diseases & Allergy, Newcastle upon Tyne Hospitals NHS Foundation Trust, Newcastle upon Tyne, United Kingdom.

^3^NIHR Newcastle Biomedical Research Centre based at Newcastle upon Tyne Hospitals NHS Trust and Newcastle University, Westgate Rd, Newcastle upon Tyne NE4 5PL, United Kingdom

^4^Newcastle University Business School, Centre for Knowledge, Innovation, Technology and Enterprise (KITE), Newcastle upon Tyne, United Kingdom

^5^Great North Children’s Hospital, Research Unit, Newcastle upon Tyne Hospitals NHS Foundation Trust, Newcastle upon Tyne, United Kingdom.

^6^Great North Children’s Hospital, Paediatric Oncology, Newcastle upon Tyne Hospitals NHS Foundation Trust, Newcastle upon Tyne, United Kingdom.

^7^Population Health Sciences Institute, Newcastle University, Newcastle upon Tyne, UK

^8^Great North Children’s Hospital, Paediatric Intensive Care Unit, Newcastle upon Tyne Hospitals NHS Foundation Trust, Newcastle upon Tyne, United Kingdom.

^9^Northumbria University, Newcastle upon Tyne, United Kingdom.

**PARTNER: LMU Munich (Germany)**

Principal Investigator

Ulrich von Both^1,2^ MD, FRCPCH (all activities)

Research group

Laura Kolberg¹ MSc (all activities)

Manuela Zwerenz¹ MSc, Judith Buschbeck¹ PhD

Clinical recruitment partners (alphabetical order)

Christoph Bidlingmaier^3^, Vera Binder^4^, Katharina Danhauser^5^, Nikolaus Haas^10^, Matthias Griese^6^, Tobias Feuchtinger^4^, Julia Keil^9^, Matthias Kappler^6^, Eberhard Lurz^7^, Georg Muench^8^, Karl Reiter^9^, Carola Schoen^9^

¹Div. Paediatric Infectious Diseases, Hauner Children’s Hospital, University Hospital, Ludwig Maximilians University (LMU), Munich, Germany

^2^German Center for Infection Research (DZIF), Partner Site Munich, Munich, Germany

^3^Div. of General Paediatrics, ^4^Div. Paediatric Haematology & Oncology, ^5^Div. of Paediatric Rheumatology, ^6^Div. of Paediatric Pulmonology, ^7^Div. of Paediatric Gastroenterology, ^8^Neonatal Intensive Care Unit, ^9^Paediatric Intensive Care Unit Hauner Children’s Hospital, University Hospital, Ludwig Maximilians University (LMU), Munich, Germany, ^10^Department Pediatric Cardiology and Pediatric Intensive Care, University Hospital, Ludwig Maximilians University (LMU), Munich, Germany

**PARTNER: bioMérieux (France)**

Principal Investigator

François Mallet^1,2, 3^

Research Group

Karen Brengel-Pesce^1,2, 3^

Alexandre Pachot^1^

Marine Mommert^1,2^

^1^Open Innovation & Partnerships (OIP), bioMérieux S.A., Marcy l’Etoile, France

^2^Joint research unit Hospice Civils de Lyon – bioMérieux, Centre Hospitalier Lyon Sud, 165 Chemin du Grand Revoyet, 69310 Pierre-Bénite, France

^3^EA 7426 Pathophysiology of Injury-induced Immunosuppression, University of Lyon1-Hospices Civils de Lyon-bioMérieux, Hôpital Edouard Herriot, 5 Place d’Arsonval, 69437 Lyon Cedex 3, France

**PARTNER: University Medical Centre Ljubljana (Slovenia)**

Principal Investigator

Marko Pokorn^1,2,3^ MD, PhD

Research Group

Mojca Kolnik^1^ MD, Katarina Vincek^1^ MD, Tina Plankar Srovin^1^ MD, PhD, Natalija Bahovec^1^ MD, Petra Prunk^1^ MD, Veronika Osterman^1^ MD, Tanja Avramoska^1^ MD

^1^Department of Infectious Diseases, University Medical Centre Ljubljana, Japljeva 2, SI-1525 Ljubljana, Slovenia

^2^University Childrens' Hospital, University Medical Centre Ljubljana, Ljubljana, Slovenia

^3^Department of Infectious Diseases and Epidemiology, Faculty of Medicine, University of Ljubljana, Slovenia

**PARTNER: Amsterdam, Academic Medical Hospital & Sanquin Research Institute (Netherlands)**

Principal Investigator

Taco Kuijpers ^1,2^

Co-investigators

Ilse Jongerius ^2^

Recruitment team (EUCLIDS, PERFORM)

J.M. van den Berg^1^, D. Schonenberg^1^, A.M. Barendregt^1^, D. Pajkrt^1^, M. van der Kuip^1,3^, A.M. van Furth^1,3^

Students PERFORM

Evelien Sprenkeler ^2^, Judith Zandstra ^2^

Technical support PERFORM

G. van Mierlo ^2^, J. Geissler ^2^

^1^ Amsterdam University Medical Center (Amsterdam UMC), location Academic Medical Center (AMC), Dept of Pediatric Immunology, Rheumatology and Infectious Diseases, University of Amsterdam, Amsterdam, the Netherlands

^2^ Sanquin Research Institute, & Landsteiner Laboratory at the AMC, University of Amsterdam, Amsterdam, the Netherlands.

^3^ Amsterdam University Medical Center (Amsterdam UMC), location Vrije Universiteit Medical Center (VUMC), Dept of Pediatric Infectious Diseases and Immunology, Free University (VU), Amsterdam, the Netherlands (former affiliation)

## **EUCLIDS Consortium**

www.euclids-project.eu

**PARTNER: Imperial College London (United Kingdom)**

**Members of the EUCLIDS Consortium at Imperial College London (UK) Principal investigator and co-investigators**

Michael Levin (grant application, EUCLIDS Coordinator, CI)

Lachlan Coin (bioinformatics), Stuart Gormley (clinical coordination), Shea Hamilton (proteomics),

Jethro Herberg (grant application, PI), Bernardo Hourmat (project management), Clive Hoggart (statistical genomics), Myrsini Kaforou (bioinformatics), Vanessa Sancho-Shimizu (genetics), Victoria Wright (grant application, scientific coordination)

Consortium members at Imperial College

Amina Abdulla, Paul Agapow, Maeve Bartlett, Evangelos Bellos, Hariklia Eleftherohorinou, Rachel Galassini, David Inwald, Meg Mashbat, Stephanie Menikou, Sobia Mustafa, Simon Nadel, Rahmeen Rahman, Hannah Shailes, Clare Thakker

**EUCLIDS UK Clinical Network**

Poole Hospital NHS Foundation Trust, Poole: Dr S Bokhandi (PI), Sue Power, Heather Barham

Cambridge University Hospitals NHS Trust, Cambridge: Dr N Pathan (PI), Jenna Ridout, Deborah White, Sarah Thurston

University Hospital Southampton, Southampton: Prof S Faust (PI), Dr S Patel (co-investigator), Jenni McCorkell.

Nottingham University Hospital NHS Trust: Dr P Davies (PI), Lindsey Crate, Helen Navarra, Stephanie Carter

University Hospitals of Leicester NHS Trust, Leicester: Dr R Ramaiah (PI), Rekha Patel

Portsmouth Hospitals NHS Trust, London: Dr Catherine Tuffrey (PI), Andrew Gribbin, Sharon McCready

Great Ormond Street Hospital, London: Dr Mark Peters (PI), Katie Hardy, Fran Standing, Lauren O’Neill, Eugenia Abelake

King’s College Hospital NHS Foundation Trust, London; Dr Akash Deep (PI), Eniola Nsirim

Oxford University Hospitals NHS Foundation Trust, Oxford Prof A Pollard (PI), Louise Willis, Zoe Young

Kettering General Hospital NHS Foundation Trust, Kettering: Dr C Royad (PI), Sonia White

Central Manchester NHS Trust, Manchester: Dr PM Fortune (PI), Phil Hudnott

**PARTNER: SERGAS (Spain)**

**Principal** **Investigators**

Federico Martinón-Torres^1^

Antonio Salas^1,2^

GENVIP RESEARCH GROUP (in alphabetical order):

Fernando Álvez González^1^, Ruth Barral-Arca^1,2^, Miriam Cebey-López^1^, María José Curras-Tuala^1,2^, Natalia García^1^, Luisa García Vicente^1^, Alberto Gómez-Carballa^1,2^, Jose Gómez Rial^1^, Andrea Grela Beiroa^1^, Antonio Justicia Grande^1^, Pilar Leboráns Iglesias^1^ , Alba Elena Martínez Santos^1^, Federico Martinón -Torres^1^, Nazareth Martinón-Torres^1^, José María Martinón Sánchez^1^, Beatriz Morillo Gutiérrez^1^, Belén Mosquera Pérez^1^, Pablo Obando Pacheco^1^, Jacobo Pardo-Seco^1,2^, Sara Pischedda^1,2^, Irene Rivero-Calle^1^, Carmen Rodríguez-Tenreiro^1^, Lorenzo Redondo-Collazo^1^, Antonio Salas Ellacuriaga^1,2^, Sonia Serén Fernández^1^, María del Sol Porto Silva^1^, Ana Vega^1,3,^ Lucía Vilanova Trillo^1^.

^1^ Translational Pediatrics and Infectious Diseases, Pediatrics Department, Hospital Clínico Universitario de Santiago, Santiago de Compostela, Spain, and GENVIP Research Group (www.genvip.org), Instituto de Investigación Sanitaria de Santiago, Galicia, Spain.

1. Unidade de Xenética, Departamento de Anatomía Patolóxica e Ciencias Forenses, Instituto de Ciencias Forenses, Facultade de Medicina, Universidade de Santiago de Compostela, and GenPop Research Group, Instituto de Investigaciones Sanitarias (IDIS), Hospital Clínico Universitario de Santiago, Galicia, Spain
2. Fundación Pública Galega de Medicina Xenómica, Servizo Galego de Saúde (SERGAS), Instituto de Investigaciones Sanitarias (IDIS), and Grupo de Medicina Xenómica, Centro de Investigación Biomédica en Red de Enfermedades Raras (CIBERER), Universidade de Santiago de Compostela (USC), Santiago de Compostela, Spain

EUCLIDS SPANISH CLINICAL NETWORK:

Susana Beatriz Reyes^1^, María Cruz León León^1^, Álvaro Navarro Mingorance^1^, Xavier Gabaldó Barrios^1^, Eider Oñate Vergara^2^, Andrés Concha Torre^3^, Ana Vivanco^3^, Reyes Fernández^3^, Francisco Giménez Sánchez^4^, Miguel Sánchez Forte^4^, Pablo Rojo^5^, J.Ruiz Contreras^5^, Alba Palacios ^5^, Cristina Epalza Ibarrondo^5^, Elizabeth Fernández Cooke^5^, Marisa Navarro^6^, Cristina Álvarez Álvarez^6^, María José Lozano^6^, Eduardo Carreras^7^, Sonia Brió Sanagustín^7^, Olaf Neth^8^, Mª del Carmen Martínez Padilla^9^, Luis Manuel Prieto Tato^10^, Sara Guillén^10^, Laura Fernández Silveira^11^, David Moreno^12^.

1. Hospital Clínico Universitario Virgen de la Arrixaca; Murcia, Spain.
2. Hospital de Donostia; San Sebastián, Spain.
3. Hospital Universitario Central de Asturias; Asturias, Spain.
4. Complejo Hospitalario Torrecárdenas; Almería, Spain.
5. Hospital Universitario 12 de Octubre; Madrid, Spain.
6. Hospital General Universitario Gregorio Marañón; Madrid, Spain.
7. Hospital de la Santa Creu i Sant Pau; Barcelona, Spain.
8. Hospital Universitario Virgen del Rocío; Sevilla, Spain.
9. Complejo Hospitalario de Jaén; Jaén, Spain.
10. Hospital Universitario de Getafe; Madrid, Spain.
11. Hospital Universitario y Politécnico de La Fe; Valencia, Spain.
12. Hospital Regional Universitario Carlos Haya; Málaga, Spain.

**Members of the Pediatric Dutch Bacterial Infection Genetics (PeD-BIG) network (the Netherlands)**

*Steering committee:*

**Coordination:** R. de Groot ^1^, A.M. Tutu van Furth ^2^, M. van der Flier ^1^

**Coordination Intensive Care**: N.P. Boeddha ^3^, G.J.A. Driessen ^3^, M. Emonts ^3, 4, 5^, J.A. Hazelzet ^3^

**Other members**: T.W. Kuijpers ^7^, D. Pajkrt ^7^, E.A.M. Sanders ^6^ , D. van de Beek ^8^, A. van der Ende ^8^

**Trial coordinator**: H.L.A. Philipsen ^1^

**Local investigators (in alphabetical order)**

A.O.A. Adeel ^9^, M.A. Breukels ^10^, D.M.C. Brinkman ^11^, C.C.M.M. de Korte ^12^, E. de Vries ^13^ , W.J. de Waal ^15^, R. Dekkers ^15^, A. Dings-Lammertink ^16^ , R.A. Doedens ^17^, A.E. Donker ^18^, M. Dousma^19^, T.E. Faber ^20^, G.P.J.M. Gerrits^21^, J.A.M. Gerver ^22^, J. Heidema ^23^, J. Homan-van der Veen ^24^, M.A.M. Jacobs ^25^, N.J.G. Jansen ^6^, P. Kawczynski ^26^, K. Klucovska ^27^, M.C.J. Kneyber ^28^, Y. Koopman-Keemink ^29^, V.J. Langenhorst ^30^, J. Leusink ^31^, B.F. Loza ^32^, I.T. Merth ^33^, C.J. Miedema ^34^, C. Neeleman ^1^, J.G. Noordzij ^35^, C.C. Obihara ^36^ , A.L.T. van Overbeek – van Gils ^37^, G.H. Poortman ^38^,S.T. Potgieter ^39^, J. Potjewijd ^40^, P.P.R. Rosias ^41^, T. Sprong ^21^, G.W. ten Tussher ^42^, B.J. Thio ^43^, G.A. Tramper-Stranders ^44^, M. van Deuren ^1^, H. van der Meer ^2^, A.J.M. van Kuppevelt ^45^, A.M. van Wermeskerken ^46^, W.A. Verwijs ^47^, T.F.W. Wolfs ^4^.

1. Radboud University Medical Center – Amalia Children’s Hospital, Nijmegen, The Netherlands
2. Vrije Universiteit University Medical Center, Amsterdam, The Netherlands
3. Erasmus Medical Center – Sophia Children’s Hospital, Rotterdam, The Netherlands
4. Translational and Clinical Research Institute, Newcastle University, Newcastle upon Tyne, United Kingdom
5. Paediatric Infectious Diseases and Immunology Department, Newcastle upon Tyne Hospitals Foundation Trust, Great North Children's Hospital, Newcastle upon Tyne, United Kingdom
6. University Medical Center Utrecht – Wilhelmina Children’s Hospital, Utrecht, The Netherlands
7. Academic Medical Center – Emma Children’s Hospital, University of Amsterdam, Amsterdam, The Netherlands
8. Academic Medical Center, University of Amsterdam, Amsterdam, The Netherlands
9. Kennemer Gasthuis, Haarlem, The Netherlands
10. Elkerliek Hospital, Helmond, The Netherlands
11. Alrijne Hospital, Leiderdorp, The Netherlands
12. Beatrix Hospital, Gorinchem, The Netherlands
13. Jeroen Bosch Hospital, ‘s-Hertogenbosch, The Netherlands
14. Diakonessenhuis, Utrecht, The Netherlands
15. Maasziekenhuis Pantein, Boxmeer, The Netherlands
16. Gelre Hospitals, Zutphen, The Netherlands
17. Martini Hospital, Groningen, The Netherlands
18. Maxima Medical Center, Veldhoven, The Netherlands
19. Gemini Hospital, Den Helder, The Netherlands
20. Medical Center Leeuwarden, Leeuwarden, The Netherlands
21. Canisius-Wilhelmina Hospital, Nijmegen, The Netherlands
22. Rode Kruis Hospital, Beverwijk, The Netherlands
23. St. Antonius Hospital, Nieuwegein, The Netherlands
24. Deventer Hospital, Deventer, The Netherlands
25. Slingeland Hospital, Doetinchem, The Netherlands
26. Refaja Hospital, Stadskanaal, The Netherlands
27. Bethesda Hospital, Hoogeveen, The Netherlands
28. University Medical Center Groningen, Beatrix Children’s hospital, Groningen, The Netherlands
29. Haga Hospital – Juliana Children’s Hospital, Den Haag, The Netherlands
30. Isala Hospital, Zwolle, The Netherlands
31. Bernhoven Hospital, Uden, The Netherlands
32. VieCuri Medical Center, Venlo, The Netherlands
33. Ziekenhuisgroep Twente, Almelo-Hengelo, The Netherlands
34. Catharina Hospital, Eindhoven, The Netherlands
35. Reinier de Graaf Gasthuis, Delft, The Netherlands
36. ETZ Elisabeth, Tilburg, The Netherlands
37. Scheper Hospital, Emmen, The Netherlands
38. St. Jansdal Hospital, Hardewijk, The Netherlands
39. Laurentius Hospital, Roermond, The Netherlands
40. Isala Diaconessenhuis, Meppel, The Netherlands
41. Zuyderland Medical Center, Sittard-Geleen, The Netherlands
42. Westfriesgasthuis, Hoorn, The Netherlands
43. Medisch Spectrum Twente, Enschede, The Netherlands
44. St. Franciscus Gasthuis, Rotterdam, The Netherlands
45. Streekziekenhuis Koningin Beatrix, Winterswijk, The Netherlands
46. Flevo Hospital, Almere, The Netherlands
47. Zuwe Hofpoort Hospital, Woerden, The Netherlands

***Swiss Pediatric Sepsis Study***

***Steering Committee****: Luregn J Schlapbach, MD, FCICM* *^1,2,3^, Philipp Agyeman, MD* *^1^,* *Christoph Aebi, MD ^1^, Christoph Berger, MD ^1^*

Luregn J Schlapbach, MD, FCICM ^1,2,3^, Philipp Agyeman, MD ^1^, Christoph Aebi, MD ^1^, Eric Giannoni, MD ^4,5^, Martin Stocker, MD ^6^, Klara M Posfay-Barbe, MD ^7^, Ulrich Heininger, MD ^8^, Sara Bernhard-Stirnemann, MD ^9^, Anita Niederer-Loher, MD ^10^, Christian Kahlert, MD ^10^, Paul Hasters, MD ^11^, Christa Relly, MD ^12^, Walter Baer, MD^13^, Christoph Berger, MD ^12^ **for the Swiss Pediatric Sepsis Study**

1. Department of Pediatrics, Inselspital, Bern University Hospital, University of Bern, Switzerland
2. Paediatric Critical Care Research Group, Mater Research Institute, University of Queensland, Brisbane, Australia
3. Paediatric Intensive Care Unit, Lady Cilento Children’s Hospital, Children’s Health Queensland, Brisbane, Australia
4. Service of Neonatology, Lausanne University Hospital, Lausanne, Switzerland
5. Infectious Diseases Service, Lausanne University Hospital, Lausanne, Switzerland
6. Department of Pediatrics, Children’s Hospital Lucerne, Lucerne, Switzerland
7. Pediatric Infectious Diseases Unit, Children’s Hospital of Geneva, University Hospitals of Geneva, Geneva, Switzerland
8. Infectious Diseases and Vaccinology, University of Basel Children’s Hospital, Basel, Switzerland
9. Children’s Hospital Aarau, Aarau, Switzerland
10. Division of Infectious Diseases and Hospital Epidemiology, Children’s Hospital of Eastern Switzerland St. Gallen, St. Gallen, Switzerland
11. Department of Neonatology, University Hospital Zurich, Zurich, Switzerland
12. Division of Infectious Diseases and Hospital Epidemiology, and Children’s Research Center, University Children’s Hospital Zurich, Switzerland
13. Children’s Hospital Chur, Chur, Switzerland

**PARTNER: Liverpool (United Kingdom)**

**Principal Investigators**

Enitan D Carrol^1^

Stéphane Paulus ^1,2^

ALDER HEY SERIOUS PAEDIATRIC INFECTION RESEARCH GROUP (ASPIRE)

(in alphabetical order):

Hannah Frederick^3^, Rebecca Jennings^3^, Joanne Johnston^3^, Rhian Kenwright^3^

^1^ Department of Clinical Infection, Microbiology and Immunology, University of Liverpool Institute of Infection, Veterinary and Ecological Sciences , Liverpool, England

1. Alder Hey Children’s Hospital, Department of Infectious Diseases, Eaton Road, Liverpool, L12 2AP
2. Alder Hey Children’s Hospital, Clinical Research Business Unit, Eaton Road, Liverpool, L12 2AP

**PARTNER: Micropathology Ltd (United Kingdom)**

Colin G Fink^1,2^, Elli Pinnock^1^

^1^Micropathology Ltd Research and Diagnosis

^2^University of Warwick

**PARTNER: Newcastle (United Kingdom)**

**Principle Investigator**

Marieke Emonts^1,2,3^

Co-Investigator

Rachel Agbeko^1,4^

1. Institute of Cellular Medicine, Newcastle University, Newcastle upon Tyne, United Kingdom
2. Paediatric Infectious Diseases and Immunology Department, Newcastle upon Tyne Hospitals Foundation Trust, Great North Children's Hospital, Newcastle upon Tyne, United Kingdom
3. Institute of Cellular Medicine, Newcastle University, Newcastle upon Tyne, United Kingdom
4. NIHR Newcastle Biomedical Research Centre based at Newcastle upon Tyne Hospitals NHS Trust and Newcastle University, Westgate Rd, Newcastle upon Tyne NE4 5PL, United Kingdom

**PARTNER: Gambia**

Suzanne Anderson: Principal Investigator and West African study oversight:

Fatou Secka: Clinical research fellow and study co-ordinator

Additional Gambia site team (consortium members):

Kalifa Bojang: co-PI

Isatou Sarr: Senior laboratory technician

Ngange Kebbeh: Junior laboratory technician

Gibbi Sey: lead research nurse Medical Research Council Clinic

Momodou Saidykhan: lead research nurse Edward Francis Small Teaching Hospital

Fatoumata Cole: Data manager

Gilleh Thomas: Data manager

Martin Antonio: Local collaborator

**PARTER: Austria**

**PI:** Werner Zenz^1^

**Co-Investigators/Steering committee:**

Daniela S. Kohlfürst^1^, Alexander Binder^1^, Nina A. Schweintzger^1^, Manfred Sagmeister^1^

^1^University Clinic of Paediatrics and Adolescent Medicine, Department of General Paediatrics, Medical University Graz, Austria

**Austrian network, participating centres in Austria, Germany, Italy, Serbia, Lithuania, patient recruitment (in alphabetical order):**

Hinrich Baumgart^1^, Markus Baumgartner^2^, Uta Behrends^3^, Ariane Biebl^4^, Robert Birnbacher^5^, Jan-Gerd Blanke^6^, Carsten Boelke^7^, Kai Breuling^3^, Jürgen Brunner^8^, Maria Buller^9^, Peter Dahlem^10^, Beate Dietrich^11^, Ernst Eber^12^, Johannes Elias^13^, Josef Emhofer^2^, Rosa Etschmaier^14^, Sebastian Farr^15^, Ylenia Girtler^16^, Irina Grigorow^17^, Konrad Heimann^18^, Ulrike Ihm^19^, Zdenek Jaros^20^, Hermann Kalhoff^21^, Wilhelm Kaulfersch^22^, Christoph Kemen^23^, Nina Klocker^24^, Bernhard Köster^25^, Benno Kohlmaier^26^, Eleni Komini^27^, Lydia Kramer^3^, Antje Neubert^28^, Daniel Ortner^29^, Lydia Pescollderungg^16^, Klaus Pfurtscheller^30^, Karl Reiter^31^, Goran Ristic^32^, Siegfried Rödl^30^, Andrea Sellner^26^, Astrid Sonnleitner^26^, Matthias Sperl^33^, Wolfgang Stelzl^34^, Holger Till^1^, Andreas Trobisch^26^ , Anne Vierzig^35^, Ulrich Vogel^12^, Christina Weingarten^36^, Stefanie Welke^37^, Andreas Wimmer^38^, Uwe Wintergerst^39^, Daniel Wüller^40^, Andrew Zaunschirm^41^, Ieva Ziuraite^42^, Veslava Žukovskaja^42^

^1^Department of Pediatric and Adolescence Surgery, Division of General Pediatric Surgery, Medical University Graz, Austria

^2^Department of Pediatrics, General Hospital of Steyr, Austria

^3^Department of Pediatrics/Department of Pediatric Surgery, Technische Universität München (TUM), Munich, Germany

^4^Department of Pediatrics, Kepler University Clinic, Medical Faculty of the Johannes Kepler University, Linz, Austria

^5^Department of Pediatrics and Adolesecent Medicine LKH Villach, Austria

^6^Department of Pediatrics and Adolescent Medicine and Neonatology, Hospital Ludmillenstift, Meppen, Germany

^7^Hospital for Children's and Youth Medicine, Oberschwabenklinik, Ravensburg, Germany

^8^Department of Pediatrics, Medical University Innsbruck, Austria

^9^Clinic for Paediatrics and Adolescents Medicine, Sana Hanse-Klinikum Wismar, Germany

^10^Departement of Pediatrics, Medical Center Coburg, Germany

^11^University Medicine Rostock, Department of Pediatrics (UKJ), Rostock, Germany

^12^Department of Pulmonology, Medical University Graz, Austria

^13^Institute for Hygiene and Microbiology, University of Würzburg, Germany

^14^Clinical Institute of Medical and Chemical Laboratory Diagnostics, Medical University Graz, Austria

^15^Department of Pediatric Orthopedics and Adult Foot and Ankle Surgery, Orthopedic Hospital Speising, Vienna, Austria

^16^Department of Paediatrics, Regional Hospital Bolzano, Italy

^17^Department of Pediatrics and Adolescent Medicine, General Hospital Hochsteiermark/Leoben, Austria

^18^Department of Neonatology and Paediatric Intensive Care, Children's University Hospital, RWTH Aachen, Germany

^19^Paediatric Intensive Care Unit, Department of Paediatric Surgery, Donauspital Vienna, Austria

^20^Department of Pediatrics, General Public Hospital, Zwettl, Austria

^21^Pediatric Clinic Dortmund, Germany

^22^Department of Pediatrics and Adolescent Medicine, Klinikum Klagenfurt am Wörthersee, Klagenfurt, Austria

^23^Catholic Children's Hospital Wilhelmstift, Department of Pediatrics, Hamburg, Germany

^24^Department of Pediatrics, Krankenhaus Dornbirn, Austria

^25^Children’s Hospital Luedenscheid, Maerkische Kliniken, Luedenscheid, Germany

^26^Department of General Paediatrics, Medical University Graz, Austria

^27^Department of Paediatrics, Schwarzwald-Baar-Hospital, Villingen-Schwenningen, Germany

^28^Department of Paediatrics and Adolescents Medicine, University Hospital Erlangen, Germany

^29^Department of Pediatrics and Adolescent Medicine, Medical University of Salzburg, Austria

^30^Paediatric Intensive Care Unit, Medical University Graz, Austria

^31^Dr. von Hauner Children's Hospital, Ludwig-Maximilians- Universitaet, Munich, Germany

^32^Mother and Child Health Care Institute of Serbia, Belgrade, Serbia

^33^Department of Pediatric and Adolescence Surgery, Division of Pediatric Orthopedics, Medical University Graz, Austria

^34^Department of Pediatrics, Academic Teaching Hospital, Landeskrankenhaus Feldkirch, Austria

^35^University Children’s Hospital, University of Cologne, Germany

^36^Department of Pediatrics and Adolescent Medicine Wilheminenspital, Vienna, Austria

^37^Department of Pediatric Surgery, Municipal Hospital Karlsruhe, Germany

^38^Hospital of the Sisters of Mercy Ried, Department of Pediatrics and Adolescent Medicine, Ried, Austria

^39^Hospital St. Josef, Braunau, Austria

^40^Christophorus Kliniken Coesfeld Clinic for Pediatrics, Coesfeld, Germany

^41^Department of Paediatrics, University Hospital Krems, Karl Landsteiner University of Health Sciences, Krems, Austria

^42^Children‘s Hospital, Affiliate of Vilnius University Hospital Santariskiu Klinikos, Lithuania

**PARTNER: Singapore**

**PI:** Martin L. Hibberd^1,2^

**Co-Investigators:**

Sonia Davila ^1^

^1^Genome Institute of Singapore, Infectious Diseases, 60 Biopolis Street, 138672 Singapore

^2^Faculty of Infectious and Tropical Disease, London School of Hygiene and Tropical Medicine, London, UK

**PARTNER: Italy**

**PI:** Isabel Delany^1^

^1^ Novartis Vaccines and Diagnostics, Via Fiorentina 1, 53100 Siena, Italy

**References**

1. Martinon-Torres F, Salas A, Rivero-Calle I, et al. Life-threatening infections in children in Europe (the EUCLIDS Project): a prospective cohort study. Lancet Child Adolesc Health 2018;2(6):404-414. DOI: 10.1016/S2352-4642(18)30113-5.

2. Nijman RG, Oostenbrink R, Moll HA, et al. A Novel Framework for Phenotyping Children With Suspected or Confirmed Infection for Future Biomarker Studies. Front Pediatr 2021;9:688272. DOI: 10.3389/fped.2021.688272.

3. Organisation WH. Multisystem inflammatory syndrome in children and adolescents with COVID-19. 2020 (<https://www.who.int/publications/i/item/multisystem-inflammatory-syndrome-in-children-and-adolescents-with-covid-19>).

4. McCrindle BW, Rowley AH, Newburger JW, et al. Diagnosis, Treatment, and Long-Term Management of Kawasaki Disease: A Scientific Statement for Health Professionals From the American Heart Association. Circulation 2017;135(17):e927-e999. DOI: 10.1161/CIR.0000000000000484.

5. S. Lamble EB, M. Attar, D. Buck, R. Bowden, G. Lunter, D. Crook, B. El-Fahmawi & P. Piazza Improved workflows for high throughput library preparation using the transposome-based nextera system. BMC Biotechnology 2013;13(104).

6. Leek JT, Johnson WE, Parker HS, Jaffe AE, Storey JD. The sva package for removing batch effects and other unwanted variation in high-throughput experiments. Bioinformatics 2012;28(6):882-3. DOI: 10.1093/bioinformatics/bts034.

7. Love MI, Huber W, Anders S. Moderated estimation of fold change and dispersion for RNA-Seq data with DESeq2. Genome Biology 2014;15(12):550. DOI: 10.1186/s13059-014-0550-8.

8. Herberg JA, Kaforou M, Wright VJ, et al. Diagnostic Test Accuracy of a 2-Transcript Host RNA Signature for Discriminating Bacterial vs Viral Infection in Febrile Children. JAMA 2016;316(8):835-45. DOI: 10.1001/jama.2016.11236.

9. Kaforou M, Wright VJ, Oni T, et al. Detection of Tuberculosis in HIV-Infected and -Uninfected African Adults Using Whole Blood RNA Expression Signatures: A Case-Control Study. PLOS Medicine 2013;10(10):e1001538. DOI: 10.1371/journal.pmed.1001538.

10. Li HK, Kaforou, M., Rodriguez-Manzano, J., Channon-Wells, S., Moniri, A., Habgood-Coote, D., Gupta, R., Mills, E. A., Arancon, D., Lin, J., Chiu, Y., Pennisi, I., Miglietta, L., Obaray, N., Mehta, R., Herberg, J., Wright, V. J., Georgiou, P., Shallcross, L., Mentzer, A. J., Levin, M., Cooke, G., Noursadeghi, M. and Sriskandan, S. . Discovery and Validation of a 3-Gene Transcriptional Signature to Distinguish COVID-19 and Other Viral Infections from Bacterial Sepsis in Adults; A Case-Control then Observational Cohort Study. (Preprint) (Available at SSRN: <https://ssrn.com/abstract=3766286> or <http://dx.doi.org/10.2139/ssrn.3766286>).

11. Liao Y, Smyth GK, Shi W. featureCounts: an efficient general purpose program for assigning sequence reads to genomic features. Bioinformatics 2014;30(7):923-30. DOI: 10.1093/bioinformatics/btt656.

12. Anders S, Reyes A, Huber W. Detecting differential usage of exons from RNA-Seq data. Genome Res 2012;22(10):2008-17. (In eng). DOI: 10.1101/gr.133744.111.

13. Kuhn M. Building Predictive Models in R Using the caret Package. Journal of Statistical Software 2008;28(5):1 - 26. DOI: 10.18637/jss.v028.i05.

14. DeLong ER, DeLong DM, Clarke-Pearson DL. Comparing the areas under two or more correlated receiver operating characteristic curves: a nonparametric approach. Biometrics 1988;44(3):837-45. (In eng).

15. Verdoni L, Mazza A, Gervasoni A, et al. An outbreak of severe Kawasaki-like disease at the Italian epicentre of the SARS-CoV-2 epidemic: an observational cohort study. The Lancet 2020;395(10239):1771-1778. DOI: 10.1016/S0140-6736(20)31103-X.

16. Whittaker E, Bamford A, Kenny J, et al. Clinical Characteristics of 58 Children With a Pediatric Inflammatory Multisystem Syndrome Temporally Associated With SARS-CoV-2. JAMA 2020;324(3):259-269. DOI: 10.1001/jama.2020.10369.

17. Alquraini A, El Khoury J. Scavenger receptors. Curr Biol 2020;30(14):R790-r795. (In eng). DOI: 10.1016/j.cub.2020.05.051.

18. Gugliandolo A, Chiricosta L, Calcaterra V, et al. SARS-CoV-2 Infected Pediatric Cerebral Cortical Neurons: Transcriptomic Analysis and Potential Role of Toll-like Receptors in Pathogenesis. International Journal of Molecular Sciences.

19. Blighe K. PCAtools: PCAtools: Everything Principal Components Analysis. R package version 2.10.0. (<https://github.com/kevinblighe/PCAtools>).
